# Supplementary material for: Intelligent metasurface system for automatic tracking of moving targets and wireless communications based on computer vision
Source: Nat Commun. 2023 Feb 22;14:989. doi: 10.1038/s41467-023-36645-3 (PMC9947232; doi:10.1038/s41467-023-36645-3)
Supplement: Supplementary file 1 — Supplementary Information [file 41467_2023_36645_MOESM1_ESM.pdf]

Supplementary Note for

Intelligent metasurface system for automatic tracking of moving targets and wireless communications based on computer vision

Weihan Li[1], Qian Ma[1], Che Liu[1], Yunfeng Zhang[1], Xianning Wu[2], Jiawei Wang[1], Shizhao Gao[1], Tianshuo Qiu[2], Tonghao Liu[2], Qiang Xiao[1], Jiaxuan Wei[1], Ting Ting Gu[3], Zhize Zhou[3], Fashuai Li[3], Qiang Cheng[1], Lianlin Li[4], Wenxuan Tang[1,*], and Tie Jun Cui[1,*]

[1] State Key Laboratory of Millimeter Waves and Institute of Electromagnetic Space, Southeast University, Nanjing 210096, China

[2] Shaanxi Key Laboratory of Artificially-Structured Functional Materials and Devices, Air Force Engineering University, Xi'an 710051, China

[3] State Key Laboratory of CAD & CG, Zhejiang University, Hangzhou 310058, China

[4] State Key Laboratory of Advanced Optical Communication Systems and Networks, School of Electronics, Peking University, Beijing 100871, China

*To whom correspondence should be addressed. E-mails: wenxuant@seu.edu.cn, tjcui@seu.edu.cn

This supplementary information contains the following sections:

Supplementary Note 1. Collecting position information of the detected objects using an Intel RealSense Depth Camera D435i (RS-camera)

Supplementary Note 2. Field calculation for the digital programmable metasurface (DPM)

Supplementary Note 3. Directional beam manipulations

Supplementary Note 4. The measured far-field results of the *x*-polarization

Supplementary Note 5. Fast inverse design algorithm of the coding pattern

Supplementary Note 6. Structure and operating process of the YOLOv4-tiny network

Supplementary Note 7. Structure and operating process of the pre-training ANN

Supplementary Note 8. The advantages and usefulness of the proposed ANN approach

Supplementary Note 9. Design of the receiver patch antenna

Supplementary Note 10. Description of switching speed of system

Supplementary Note 11. Collection and production of data sets. The performance of target tracking algorithms and experimental results in the case of multi-object tracking (MOT), and when the target is might be temporarily blocked.

Supplementary Note 12. Description of experiments with multiple different classes of targets

Supplementary Note 13. Performance of target tracking algorithms and experimental results in the case of limited ambient light

Supplementary Note 14. Description of working mechanism of the detector AD8317

Supplementary Note 15. Detailed of outdoor test results

Supplementary Note 16. Experiments of BER test

Supplementary Note 17. Discussion on how to solve the potential issue of interference from other actively communicating devices operating at a similar frequency

Supplementary Note 18. The energy consumption of the proposed design

Supplementary Note 19. The structure of the YOLOv4-tiny network used in this paper.

Supplementary References

Supplementary Note 1. Collecting position information of the detected objects using an Intel RealSense Depth Camera D435i (RS-camera)

The actual captured range of the RS-camera is measured using the method of geometrical optics, as shown in Supplementary Fig. 1(a). First, we compared the rectangular area captured by the RS-camera at the distance of dx_1=25.7 cm. The distance of dx_1 can be set according to the test platform. We put a white background board in the detection area of the camera, and then combined with the display area of the camera on the computer, we drew the boundary of the detection area, that is, the measured lx_1=27.6 cm and ly_1=19.2 cm. According to the datasheet, the RS-camera works ideally when the object is no farther than 3 m away from the camera. So, when dx_1+dx_2=3 m, we can deduce that lx_2 and ly_2 are 3.22 m and 2.24 m respectively.

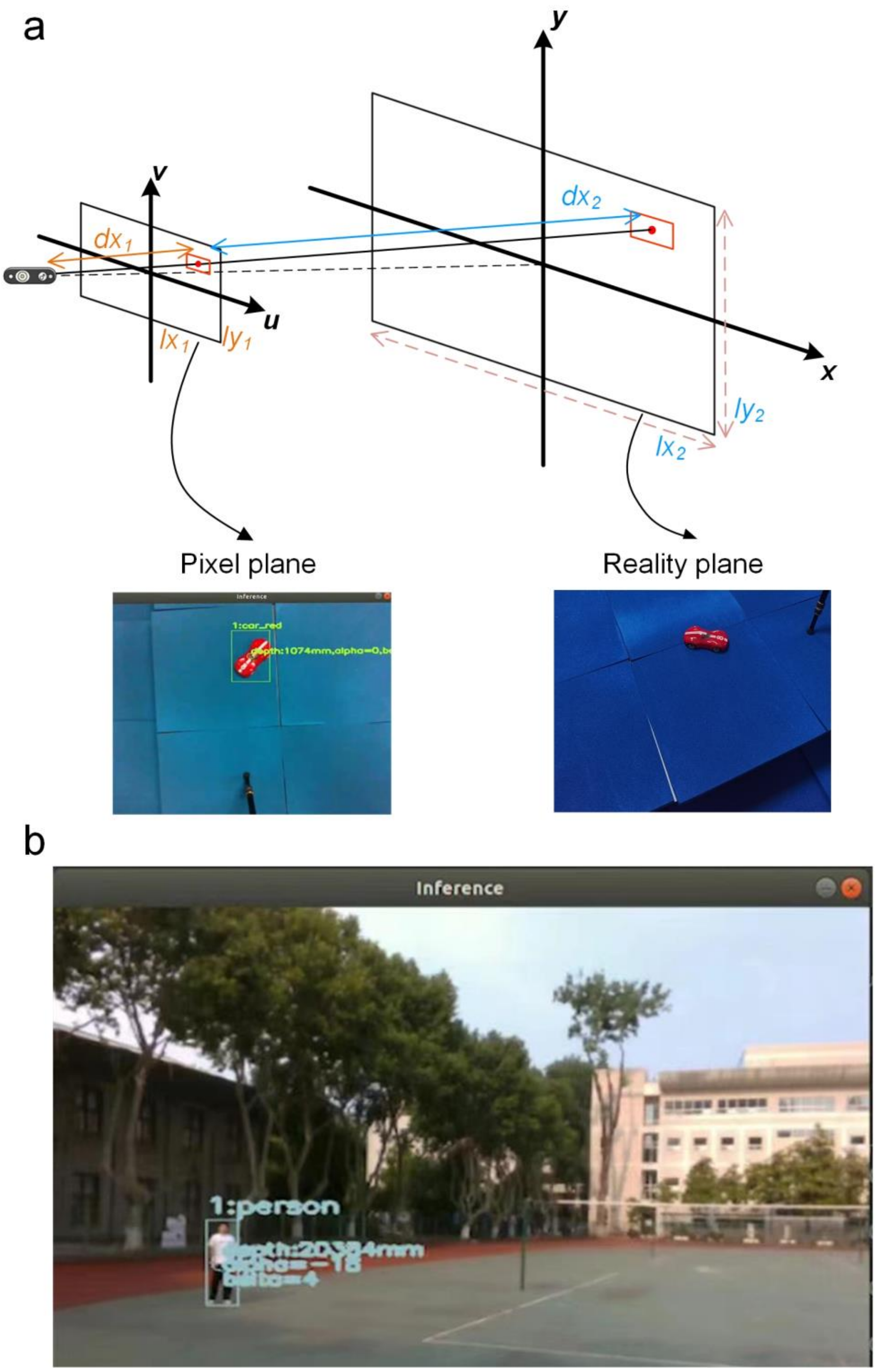

Supplementary Fig. 1 (a) Mapping diagram of pixel plane and reality plane, captured range of the RS-camera based on the geometrical optics. (b) The actual measured scene displayed on the computer. Outdoor performance of the RS-Camera, when the target is about 20m away from the RS-Camera.

Therefore, we conclude that the volume of the real scenario taken by the RS-camera is a pyramidal area, whose apex is located at the position of the camera, height is 3 m and the bottom surface is a rectangle of 3.22×2.24 m^2, as indicated in Fig. RS1(a). We calibrated the camera by processing the closer distance measurements in the manner shown in Fig. RS1(a). Fig. RS1(b)

shows the actual measured scene displayed on the computer. When the target is about 20m away from the RS-Camera, the detection task can still be completed.

Based on the pinhole imaging model, the corresponding relationship between the pixel plane and reality plane is established. Through the YOLOv4-tiny algorithm, the position of the target in the pixel coordinate system can be obtained. The azimuth angle of the target relative to the camera can be obtained by combining the field angle parameters of the camera. After obtaining the depth data of the target, one is able to multiply it with the sine values of yaw angle and pitch angle respectively to calculate the x and y position of the target in the three-dimensional reality coordinates, and multiply it with the cosine value of azimuth angle to calculate z position. The steps of calculation are as follows.

- Input parameters: w, h, u, v, V, H, d.

  w: the width of the image,

  h: the height of the image,

  (u, v): pixel coordinates of target in the image,

  V: horizontal field angle of RS-camera,

  H: vertical field angle of RS-camera,

  d: distance from the RS-camera to the target center.
- Output parameters: (x, y, z). (x, y, z): 3D coordinates of the target in the coordinate system with the RS-camera as the origin.
- Step 1: Calculate the yaw angle α and pitch angle β of the target relative to the RS-camera, $\alpha = \frac{u}{w}V,\ \beta = \frac{v}{h}H.$
- Step 2: Calculate the 3D coordinates of the target, $x = d\ sin\alpha,\ y = d\ sin\beta,\ z = d\ cos\alpha\ cos\beta.$

Supplementary Note 2. Field calculation for the digital programmable metasurface (DPM)

The scattering-field calculation for conventional metasurfaces generally depends on the phase distribution on a 2D plane (see Supplementary Fig. 2(a). For a metasurface including $N \times N$ elements, the related scattering field is calculated as [22]

$$f(\theta,\varphi) = \sum_{m=1}^{N}\sum_{n=1}^{N}\exp\left\{-i\left\{\varphi(m,n) + kD\sin\theta\left[\left(m-\frac{1}{2}\right)\cos\varphi + \left(n-\frac{1}{2}\right)\sin\varphi\right]\right\}\right\} \quad \text{(S1)}$$

where θ and φ are respectively the elevation and azimuth angles of an arbitrary direction. Relative to the metasurface, when the object is detected at a certain angle, the coding pattern on the metasurface has to be extended to a 3D version as shown in Supplementary Fig. 2(b), and the related field is $f(\theta+\Delta\theta,\varphi+\Delta\varphi)$:

$$f(\theta+\Delta\theta,\varphi+\Delta\varphi)=\sum_{m=1}^{N}\sum_{n=1}^{N}\exp\left\{-i\left\{\varphi(m,n)+kD\sin(\theta+\Delta\theta)\left[\left(m-\frac{1}{2}\right)\cos(\varphi+\Delta\varphi)+\left(n-\frac{1}{2}\right)\sin(\varphi+\Delta\varphi)\right]\right\}\right\} \quad \text{(S2)}$$

where θ and φ respectively represent the elevation and azimuth angles of the moving target relative to the 2D version. The metasurface is able to automatically adjust the coding pattern according to the spatial displacement $(\Delta\theta,\Delta\varphi)$. The YOLOv4-tiny can exactly detect the displacement of the moving target and update the position information of it, and promptly instructs the FPGA to generate the corresponding coding pattern. Thus the problem can be simplified to calculate the coding pattern for the specific scattering direction $(\Delta\theta,\Delta\varphi)$.

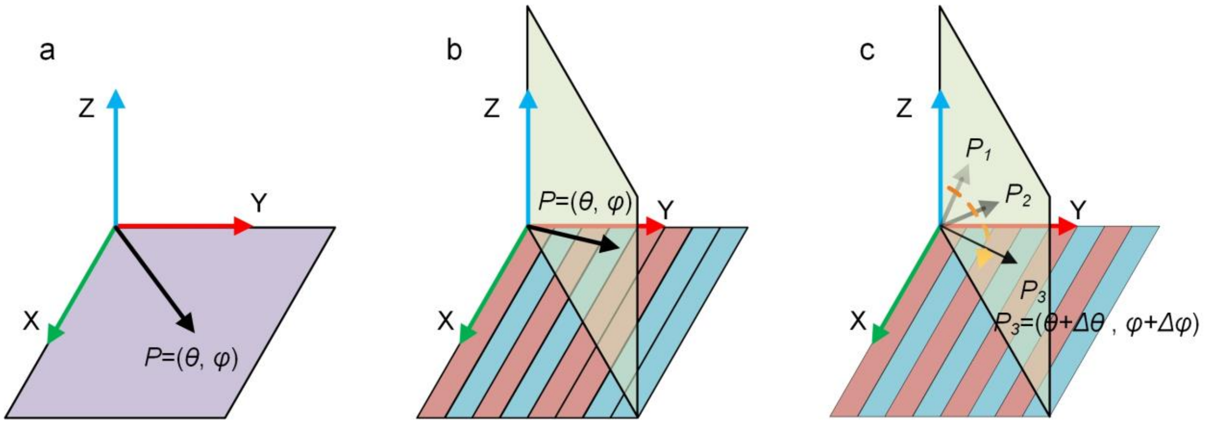

Supplementary Fig. 2 The illustration of scattering-field calculation for rotated metasurface. (a) The coding patterns for 2D versions. (b-c) The 2D far-field scattering calculations for 3D versions. P_1, P_2 and P_3 are the positions of a moving target at different time points.

Supplementary Note 3. Directional beam manipulations

A total of 324 (18 × 18) unit cells constitute the aperture of the DPMs, as shown in Supplementary Fig. 3(a). A y-polarized rectangular horn antenna is used to illuminate the DPMs. According to the superposition principle, reflection of the DPM is the superposition of reflections of all the unit cells. For simplicity, the unit cell with an ON-state diode is represented by digit “1” and the one with an OFF-state diode is represented by digit “0”. Distributions of these digits are referred as digital coding schemes of the DPMs. With adequate configuration

of the digital coding schemes, DPMs can generate beams at different deflection angles. And steerable beams are available if the digital coding schemes are dynamically configured. Supplementary Fig. 4 shows the digital coding schemes for DPMs with the beam varying from -40° to 40° and Supplementary Fig. 5 gives the the photographs of the fabricated prototype.

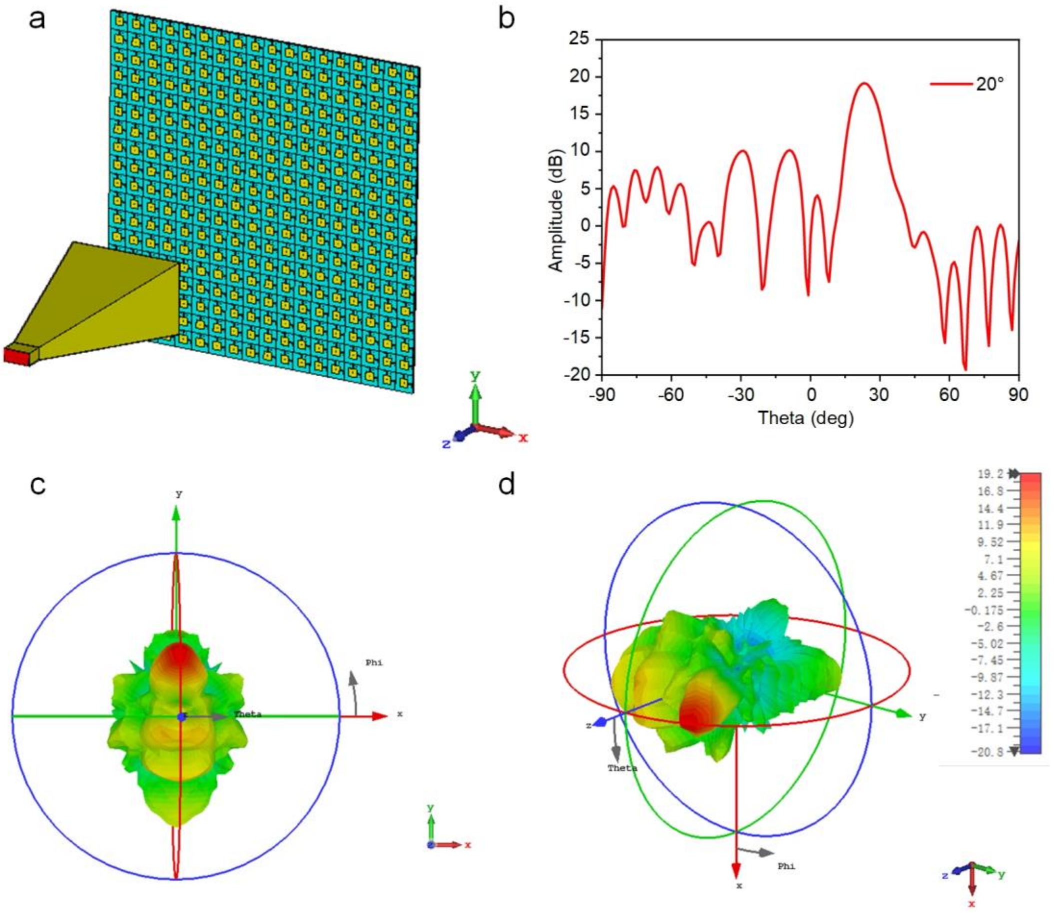

Supplementary Fig. 3 Calculations of 20° beam at 5.8 GHz. (a) Simulation model of the proposed DPM. (b) Calculated 2D far-field results. The calculated 3D far-field results in perspective of (c) and (d) when the angle is 20°.

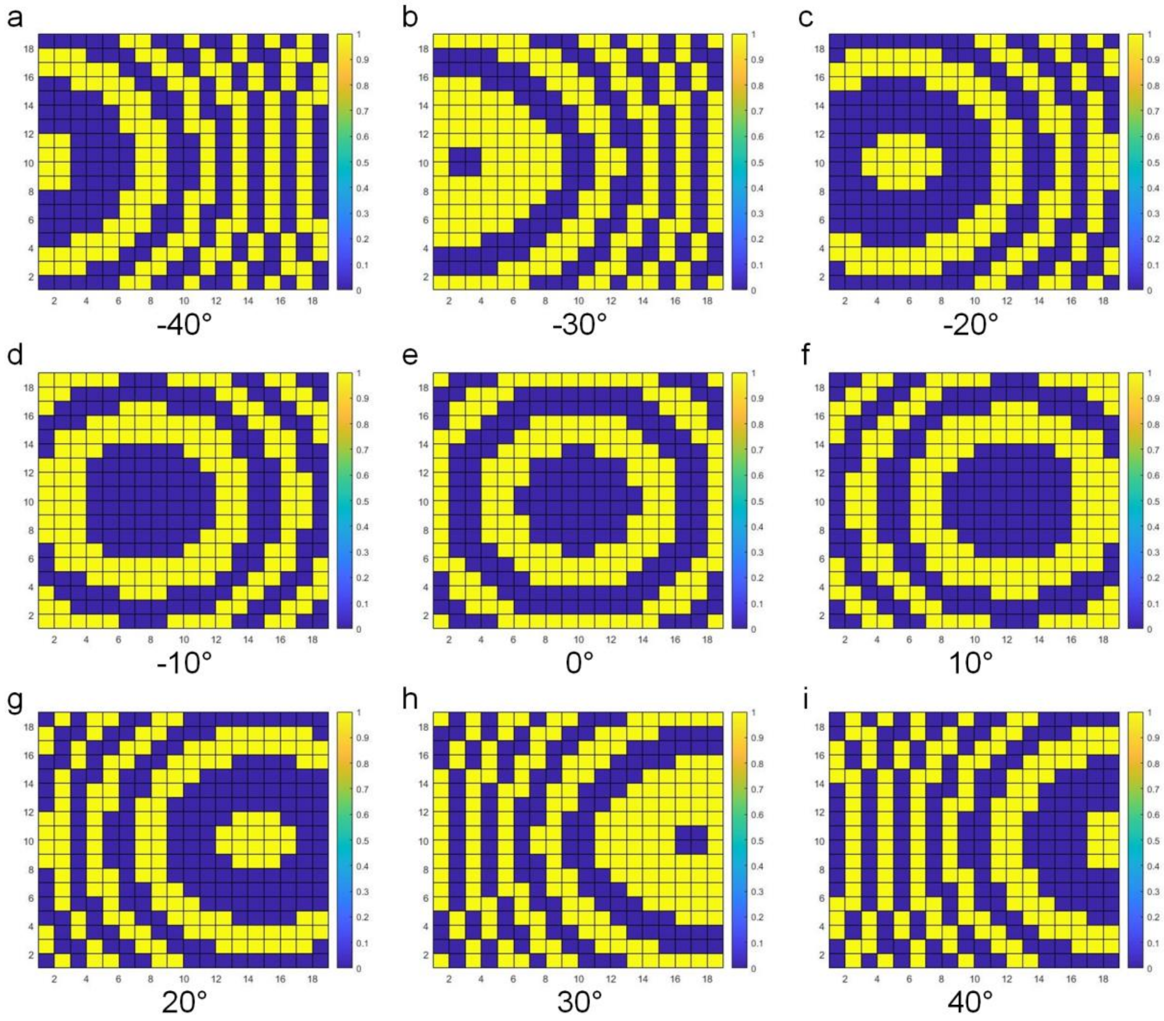

Supplementary Fig. 4 (a-i) Digital coding schemes for beams varying from -40° to 40° in the E-plane. Blue patches denote OFF-state PIN diodes, and yellow patches denote ON-state PIN diodes.

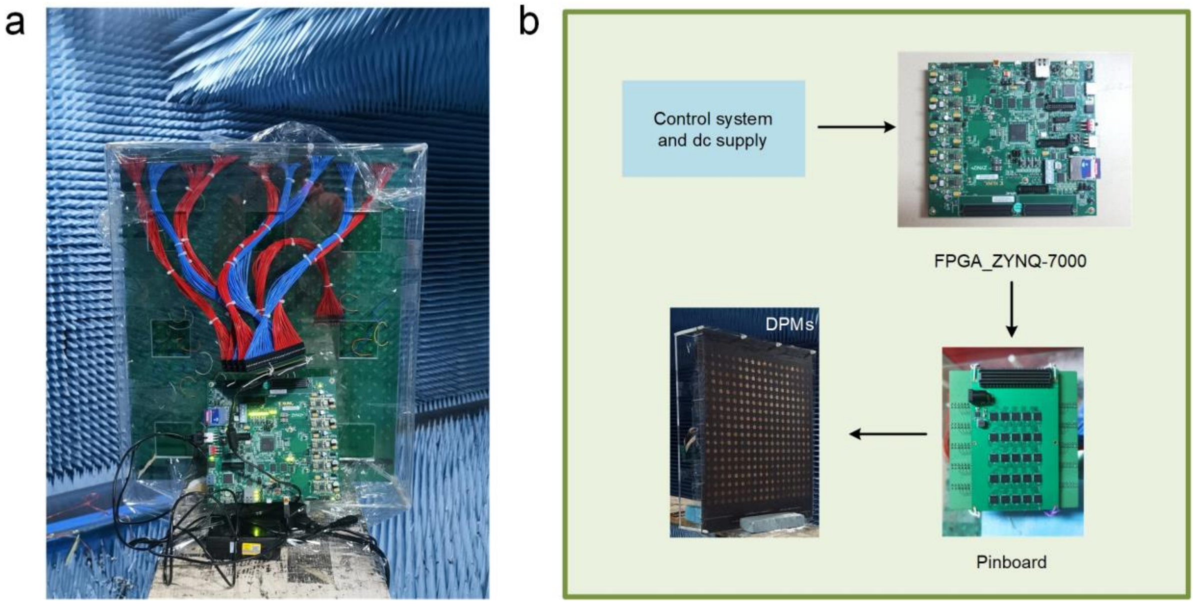

Supplementary Fig. 5 (a) Photographs of the back side of the fabricated prototype. (b) The flow chart of DPMs and feeding network. The DPMs sample has a thickness of about 4.052 mm in total, FMC output on FPGA is connected to the DPMs through a pinboard.

Supplementary Note 4. The measured far-field results of the *x*-polarization

We tested the performance of beam steering when *x*-polarized feeding horn is adopted. Supplementary Fig. 21 (a, b) show the working diagram of the feeder for each polarization. The experimental setup is shown in Supplementary Fig. 6 (c, d). The DPM presents great performance of dynamic beam scanning controlled by the FPGA, and Supplementary Fig. 6 (e) plots the measured beams on the E-plane from -40° to 40° with an increment of 10°. With the increment of the scanning angle, the gain decreases from 18.77 dB to 14.43 dB and the beam width becomes wider due to the fact that the effective aperture of the DPMs becomes smaller as the scanning angle increases. Nevertheless, the good performance of designable radiation patterns and steerable power distributions guarantees the feasibility of the proposed intelligent tracking system in both polarizations. Thus, we conclude that the working bandwidth of the metasurface can be obtained to be about 200MHz.

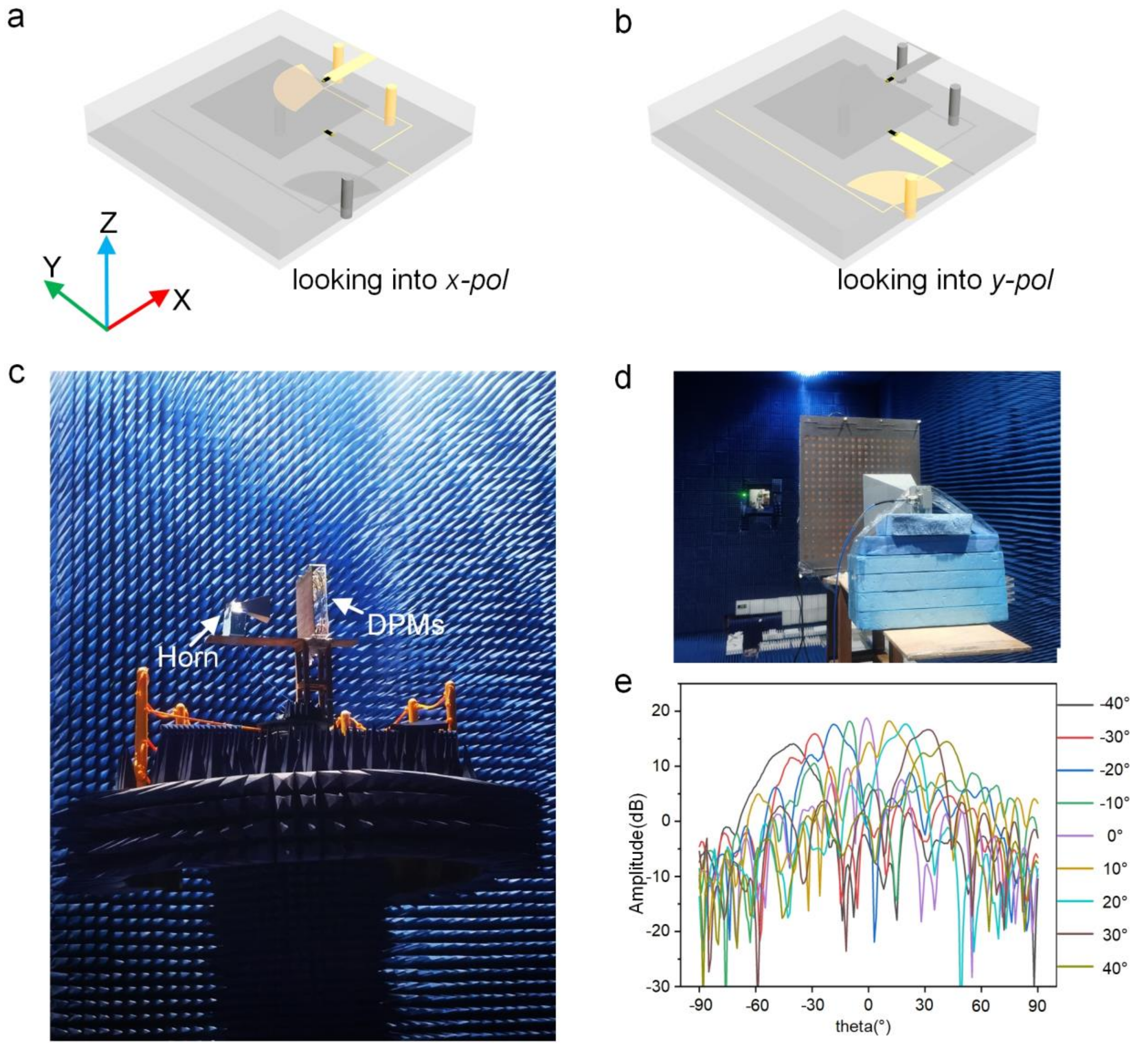

Supplementary Fig. 6 Perspective views of the coding element and (a, b) working diagram of the feeder for each polarization. (c) A *x*-polarized rectangular horn antenna is used to illuminate the DPM. The far-field experimental setup in an anechoic chamber. (d) Front view of the fabricated DPM and the experimental setup. (e) The measured far-field patterns when beams on the E-plane vary from -40° to 40° at 5.8 GHz. The experiment verifies that for *x*-polarized incidence, the DPMs can also shape the far-field patterns in the spatial domain with the steerable beam.

Supplementary Note 5. Fast inverse design algorithm of the coding pattern

For the part of vision detection and target tracking, we adopt the architecture based on YOLOv4-tiny, and combine target tracking and coordinate transformation of the scene to realize the real scene.

Target detection is an overlay of classification and regression. Early visual detection used

sliding windows of various sizes to slide on the image to select candidate regions, and manually designed the kinds of features extracted from the candidate regions to determine the presence or absence of targets and also the classes of targets in the candidate boxes. The traditional detection method is time-consuming and the manually designed features are poorly robust and difficult to cope with new scenes after transplantation. Along with the evolution of artificial intelligence and the development of high-performance computing resources, intelligent target recognition with deep learning as the core has emerged, showing unique features. Target detection algorithms based on deep learning are mainly divided into two categories: two-stage algorithms and one-stage algorithms. The typical model of two-stage algorithms is the RCNN series. The main idea is to generate multiple candidate boxes in the image based on the correlation algorithm, and extract the depth features of the candidate boxes. First, judge whether there are targets in the candidate boxes, and then classify the candidate boxes with targets. Judging the regression with the frame, the specific category of the object in the frame and the more accurate detection frame can be obtained. In contrast, a one-stage algorithm directly classifies and regresses the candidate box, and judges whether there are objects in the candidate box according to the confidence score of classification. Compared with the two-stage algorithm, it saves the step of judging whether there are objects in the candidate box. So, the one-stage algorithm is faster, but the accuracy is lower than that of the two-stage algorithm. YOLO series is a typical model in one-stage algorithm.

Visual tracking is the process of continuously locating a target in subsequent frames, given the position and size of the target in the initial frame. The process carries on continuous localization of the target in the subsequent frames. Compared with the visual detection task, the visual tracking task requires the initial position and size of the target to be determined in advance, and then use the temporal and spatial continuity and correlation of the images between frames to achieve continuous localization of the target.

The difference between the two-stage algorithm and YOLOv4-tiny for anchor frame position prediction and regression lies in that the two-stage algorithm uses the regional candidate network to get the filter box and then regression, while YOLOv4-tiny directly performs the border regression for all anchor frames. The process is shown in Supplementary Fig. 7.

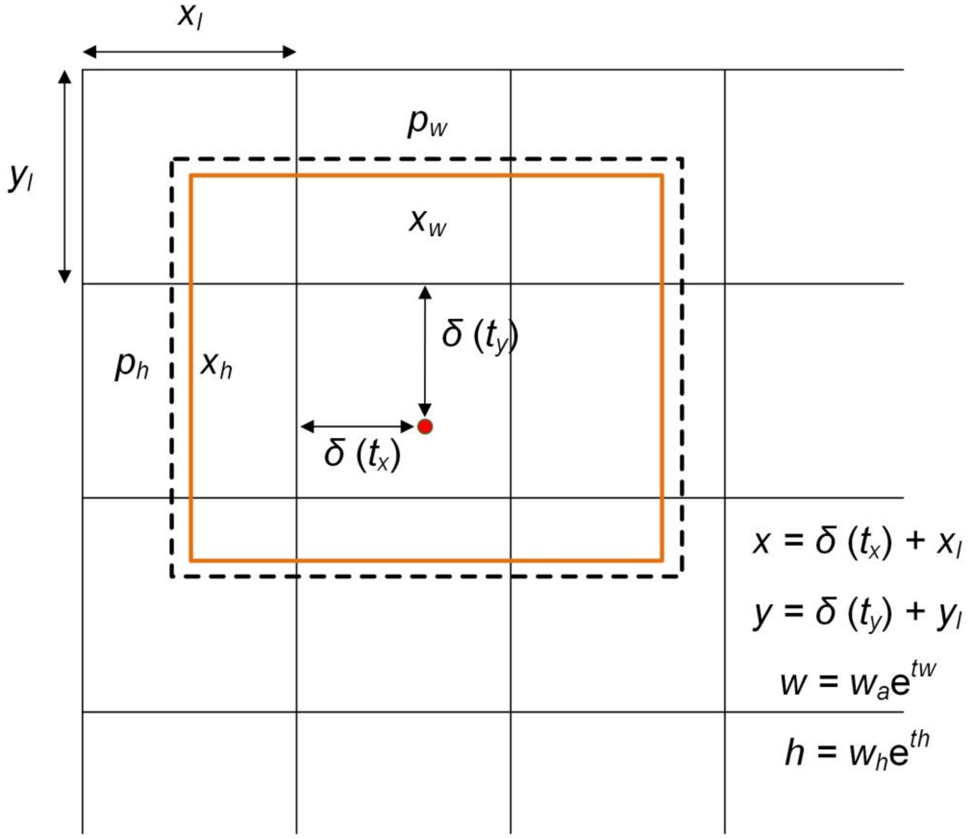

Supplementary Fig. 7 YOLO border prediction.

YOLO-v4 divides the input image into S×S regions, and only detects the anchor frame of each region, in other words, only the target whose center falls in this region. Assume that the coordinates of the upper left corner of the area corresponding to [x_l, y_l], and the width and height are [w_a, h_a]; The center coordinate of the prediction box is [x, y], and the width and height are w and h. The real center coordinate of the real frame is t_x, and the width and height are t_w and t_h, respectively. Abscissa regression parameters of prediction frame and anchor frame t_x, ordinate regression parameter t_y, width regression parameter t_w^* and height regression parameter t_h^*. The confidence of the prediction box is t_o. The relationships between regression parameters and data are given below:

$$\delta(t_x) = x - x_l, \delta(t_y) = y - y_l \quad \text{(S3)}$$

$$t_w = \log\left(\frac{w}{w_a}\right), t_y = \log\left(\frac{h}{h_a}\right) \quad \text{(S4)}$$

$$\delta(t_x^*) = x^* - x_l, \delta(t_y^*) = y^* - y_l \quad \text{(S5)}$$

$$t_w^* = \log\left(\frac{w^*}{w_a}\right), t_h^* = \log\left(\frac{h^*}{h_a}\right) \quad \text{(S6)}$$

in which δ represents sigmoid normalization function, which is used to ensure that the target frame does not exceed the area where the initial anchor frame is located after regression, and is conducive to accelerating convergence. The model predicts and regresses the corresponding anchor frames in different feature maps, and finally obtains the detection results in the form of $(t_x, t_y, t_w, t_h, t_o)$, where t_o represents the probability that the detection frame is a certain target. Tolov4-tiny target detection algorithm can classify multiple targets in the field of vision at the same time, and give the categories to which the targets belong. By judging the category, the position information of the specified target is extracted, and the beam is controlled to point to the specified target.

Supplementary Note 6. Structure and operating process of the YOLOv4-tiny network

We mainly made the following four parts as supplementary explanations:

1. The reason for selecting the YOLO series in the detection algorithm.
2. Design of loss function in YOLOv4-tiny.
3. Structure of YOLOv4-tiny, see Supplementary Note 19 for details.
4. Meaning and values of some important hyper-parameters in YOLOv4-tiny.

In computer vision, there are four main types of tasks regarding image perception: **classification, localization, detection and segmentation**. Classification is responsible for determining the class of a target contained within an image, localization is responsible for determining the location of the pixels of the target, detection includes locating all targets in the image and classifying them, and segmentation requires determining the target or scene to which each pixel belongs.

Object detection is an overlay of classification and regression. In the application scenarios of this work, the location of the target needs to be obtained by the vision sensor, and the pixel coordinates and the label of the target in the image need to be obtained first. In view of this, object detection in the field of computer vision is well suited in this work.

In early visual object detection, people used sliding windows of various sizes to slide on the image to select candidate regions. Manually select which features to extracted from the candidate regions, to determine the presence or absence of targets as well as the classes of

targets in the candidate boxes, as shown in Supplementary Fig. 8. However, such detection methods are time-consuming and the manually designed features are poorly robust, difficult to cope with different scenarios.

In view of this, we adopted the YOLO (You Only Look Once) series of algorithms which is a deep learning based object detection algorithm. There are two main metrics for evaluating the complexity of object detection algorithms, FLOPs (Floating Point Operations) and parameters.

(1) Floating-point operations refer to the number of additions and multiplications performed during the inference of the model, which describes the computational power required by the network model to inference and reflects the performance requirements of the algorithm to the hardware (e.g. GPU). (2) Parameters refers to the number of convolution kernel weights, full connected layer weights and other learned weights in the neural network, which reflects the amount of memory required by the model for inference.

For a convolutional layer, the FLOPs and parameters are calculated as:

$$\text{FLOPs} = HWC_{out}\,(\mathrm{K}^2\mathrm{C}_{in} + 1) \quad \text{(S7)}$$

$$\text{Params} = C_{out}\,(\mathrm{K}^2\mathrm{C}_{in} + 1) \quad \text{(S8)}$$

where 1 denotes the bias operation, H and W are the size of the output feature map, and C_{in}, C_{out} denote the number of input and output channels, and K is the convolution kernel size[1].

The YOLO family of algorithms is currently available in several versions[2-5]. Among them, YOLOv4 and derivative algorithms are the most effective. YOLOv4 has 64.4 M parameters and 142.8 GFLOPs, while YOLOv4-tiny has only 6.1 M of parameters, ten times less than YOLOv4, and 6.9 FLOPs (note: 1 GFLOPs = 109 FLOPs). Although the mAP on the COCO dataset was 62.8% for YOLOv4 and 40.2% for YOLOv4-tiny, **the YOLOv4-tiny network with fewer parameters, faster loading and higher speed** was chosen considering our simpler experimental scenario and lower hardware performance[6]. The YOLOv4-tiny algorithm is used in this paper.

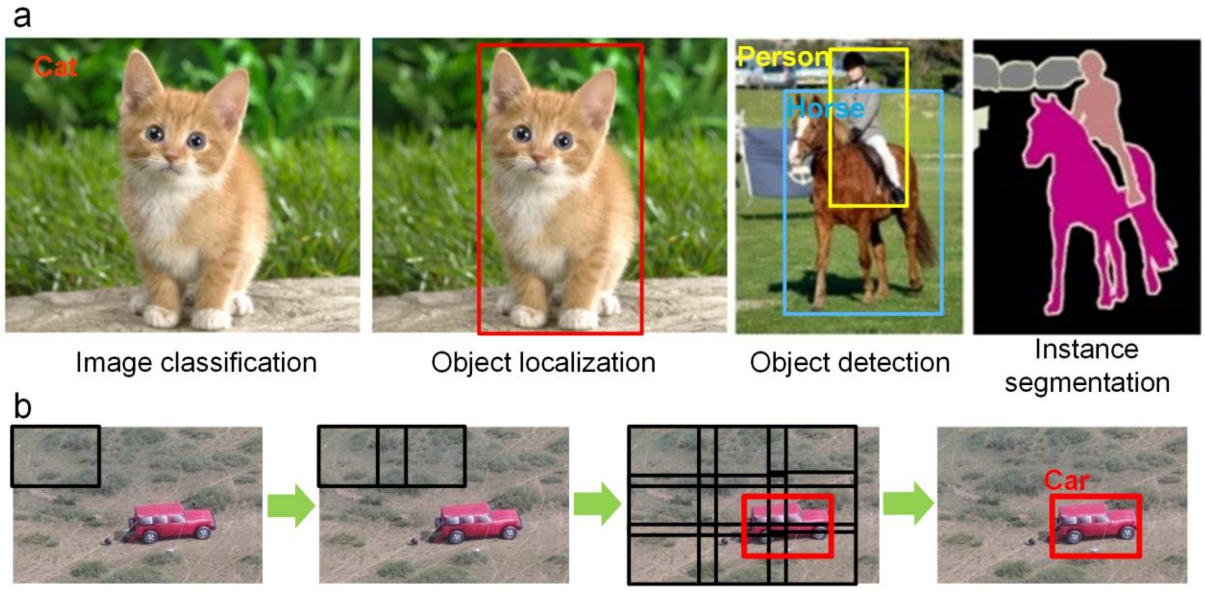

Supplementary Fig. 8 (a) Schematic representation of four main tasks in computer vision. (b) Traditional object detection method.

The YOLOv4-tiny algorithm is a supervised learning algorithm that requires images containing targets to be acquired and manually labelled in advance, and used as a dataset to train and validate the model. YOLOv4-tiny takes a colour image consisting of three channels of RGB as input, extracts features through a convolutional neural network, classifies and regresses the image features, and outputs a rectangular bounding box (x, y, w, h) containing the target, the confidence of containing the object in bounding box, and the object class (label1, label2, ..., labelN). In the training mode, all parameters within the convolutional neural network are randomly initialised, and after reasoning on the input image, the output (x, y, w, h, confidence, label1, label2, ..., labelN) is obtained and substituted into the loss function with the ground truth to find the loss value and use the gradient back-propagation using the gradient descent method, so that the network parameters converge to the optimal value. The loss function is shown in Equations (S9)-(S13).

$$L_{total} = L_{xy} + L_{wh} + L_{cof} + L_{cla} \tag{S9}$$

$$L_{xy} = \lambda_{coord} \sum_{i=0}^{S^2} \sum_{j=0}^{B} l_{ij}^{obj} [(x_i - \hat{x}_i)^2 + (y_i - \hat{y}_i)^2] \tag{S10}$$

$$L_{wh} = \lambda_{coord} \sum_{i=0}^{S^2} \sum_{j=0}^{B} l_{ij}^{obj} [(\sqrt{w_i} - \sqrt{\hat{w}_i})^2 + (\sqrt{h_i} - \sqrt{\hat{h}_i})^2] \tag{S11}$$

$$L_{cof} = \sum_{i=0}^{S^2} \sum_{j=0}^{B} l_{ij}^{obj} (c_i - \hat{c}_i)^2 + \lambda_{noobj} \sum_{i=0}^{S^2} \sum_{j=0}^{B} l_{ij}^{noobj} (c_i - \hat{c}_i)^2 \quad \text{(S12)}$$

$$L_{cla} = \sum_{i=0}^{S^2} \sum_{c \in classes} (p_i(c) - \hat{p}_i(c))^2 \quad \text{(S13)}$$

In equation (S9), L_{total} represents total loss, L_{xy} represents loss of centre, L_{wh} represents loss of width and height, L_{cof} represents loss of confidence and L_{cla} represents loss of classification. In equation (S10), S^2 indicates the number of anchor boxes, B indicates the number of prediction boxes, B=3 in YOLOv4-tiny, l_{ij}^{obj} indicates whether there is a object in the box, x and y indicates the ground truth centrecoordinate, $\hat{x}$ and $\hat{y}$ indicates the prediction box centre coordinate, λ_{coord} indicates the loss of centre coordinate weight($\lambda_{coord} = 5$). Equation (S11) where w denotes thewidth of ground truth, h denotes the height of ground truth, $\hat{w}$ denotes the width of prediction box and $\hat{h}$ denotes the height of prediction box. In equation (S12) λ_{obj} denotes the non-object weight ($\lambda_{obj} = 0.5$), c denotes whether the anchor box contains the object, i.e. $c = 1$ means the ground truth contains the object , $c = 0$ means the opposite, and $\hat{c}$ denotes whether the anchor box contains the predicted value of the target, also means the confidence. In equation (S13), $p(c)$ denotes the ground truth of the labelclassification and $\hat{p}(c)$ denotes the prediction for the labelclassification.

In this paper, the YOLOv4-tiny input data is a RGB image of dimension (608,608,3), where (608,608) is the resolution of the image and 3 refers to the colour image consisting of three channels of RGB (red, green, blue). The input data through the convolutional neural network to finally output (19,19,24), (38,38,24), where (19,19), (38,38) is the width and height of the output data, and 24 is obtained from (5+3) × 3, where × 3 means that each point on the output data will correspond to 3 prediction bounding box, and (5+3) represents the prediction box(x, y, w, h) and confidence, predicted label(car_red, car_blue, car_night), if the targetin the box is a red car, then the predicted labelis (1, 0, 0) and the blue car is (0, 1, 0), the predicted label under infrared imaging is (0,0,1).

The structure of the YOLOv4-tiny network used in this paper is shown in Supplementary Note 19. (see Supplementary Note 19 for details).

The YOLOv4-tiny model uses gradient descent to find the minimum of the loss function during training, which is iterative, meaning that the data needs to be computed several times during training to find the optimal solution. If the training data is too large for all the data to be fed into the computation at once, a small amount of data needs to be put in several times, and the amount of data to be put in each time is the batchsize. The choice of batchsize is crucial, and Supplementary Fig. 9 shows the training results when the batchsize is of different sizes. In blue, all the data is fed into the training, i.e. the batchsize contains all the training samples. Green is minibatch, i.e. all the data is divided into several batches, each containing a small number of training samples. In red, the training is random, i.e. batchsize=1. As can be seen from the diagram, the best results are obtained by putting the whole data in at once, and this is the best way to train when the amount of data is small and the computer can carry it. If you put in a small number of training samples at a time, there is a slight loss of accuracy, and if you choose a random sample for training, the model will be easily biased by the noise in the dataset, making it difficult to reach convergence. The choice of batchsize therefore also determines the accuracy of the fit of the network model to the training data.

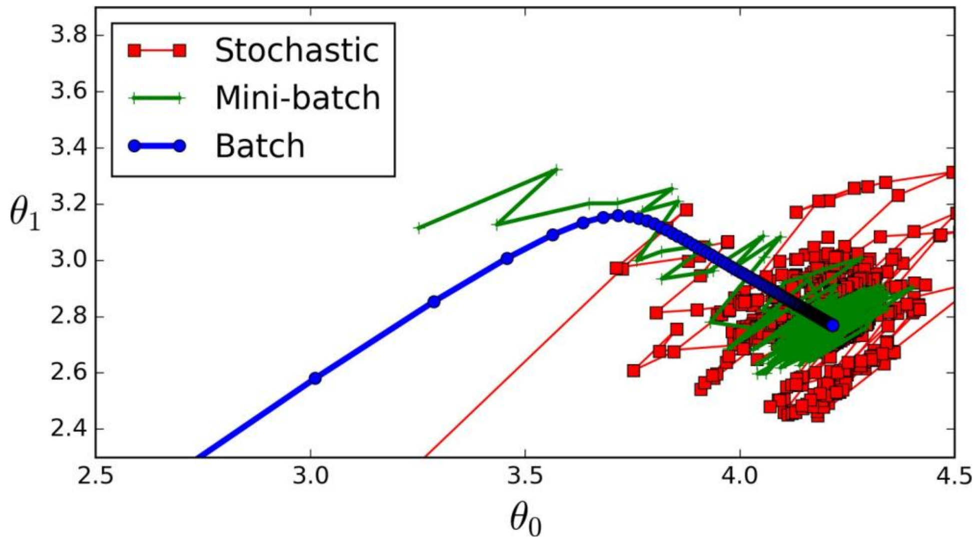

Supplementary Fig. 9 The training results when the batchsize is of different sizes[7], from Hands-On Machine Learning with Scikit-Learn and TensorFlow, by Aurélien Géron. Copyright © 2017 Aurélien Géron. Published by O'Reilly Media, Inc. Used with permission.

Within a certain range, the larger the batchsize, the more accurate the direction of descent is and the less training oscillation it causes. However, after the batchsize increases to a certain level, the determined descent direction basically does not change anymore, and instead the convergence of parameter is slowed down by the need to process too much data in one iteration.

Therefore, the parameter batch=64 in the YOLOv4-tiny paper is chosen in this paper.

For the Nvidia GTX 1650 GPU used in our system, the video memory cannot load 64 images at once, so the subdivisions parameter is used. Let subdivisions=16, which means that the video memory is loaded 4 images at a time, and the results are saved after processing until 64 images have been processed, then gradient descent is performed on their computed loss values. subdivisions ensures that algorithm training can be performed on devices with low computing power.

The max_batches parameter refers to the maximum number of iterations for training, and the algorithm stops when the number of iterations reaches max_batches. max_batches is too small and the model will stop early before it reaches the optimal parameters, while max_batches is too large and the loss function has already reached its minimum value, and further training will waste equipment resources. The number of images in the training set used in this paper is 162, so max_batches = 9999 can be used to train the model to the optimum.

It is also important to choose the step size of the gradient descent method, as too large a step size can lead to oscillations and difficulty in convergence after iterating around the optimum point, while too small a step size can lead to slow iterations and take longer to converge. Therefore, using a variable step size for parameter update, the training set has 162 data, when iterating 1300 batches, it is equivalent to 500 iterations of all the data, at this time, the update step size can be changed to 1/10 of the original one, so that it converges more finely and it is easier to reach the optimal point. Considering the number of data sets and the training speed of the YOLOv4-tiny model, the step size was reduced to 1/10 of the original size after 1300 and 1800 generations respectively in this paper.

The input size of the YOLOv4-tiny model is fixed and generally chosen as a multiple of 32. In the YOLOv4-tiny paper, two input sizes were used for experimentation; a larger input size would yield more features and higher accuracy, so the input size of 608 was chosen for this paper.

In addition to the above parameters, the hyperparameters that need to be determined for model training are momentum, decay, angle, saturation, exposure, hue, and classes. the meanings and values of the parameters are shown in Supplementary Table 1.

Supplementary Table 1. Description and value of some parameters in YOLOv4-ting

	Implications	The value of this paper
**batch**	The number of images used for each parameter update during training. When the predicted values are obtained by inference on the batch images, and the loss function and gradient are calculated, then the model parameters are updated using gradient descent.	64
**subdivisions**	When setting the parameters, the performance of the GPU is taken into account. GPUs with different performance have different video memory sizes, and GPUs with smaller video memory cannot put in batch images for training at the same time, so the batch is divided into subdivisions and put into the video memory for training, but the parameters are still updated once for each batch image.	16
**max_batches**	Maximum iterations	9999
**steps**	Learning rate change step	1300, 1800
**scales**	Learning rate change factor, when the number of iterations reaches steps, the current learning rate is multiplied by the scales as the new learning rate.	0.1, 0.1
**Width height**	Image size for network input	608, 608
**momentum**	Momentum parameters in the momentum gradient descent algorithm	0.9
**decay**	Weight decay canonical coefficients, which are used to prevent over-fitting	0.0005
**Angle**<br>**Saturation**<br>**Exposure**<br>**Hue**	Data enhancement parameters applied to the input image during training	3<br>1.5<br>1.5<br>0.1
**classes**	Number of target classes to be detected by the network	3

Supplementary Note 7. Structure and operating process of the pre-training ANN

We will first briefly introduce the nonlinear optimization algorithm with low sidelobe and then introduce the details of artificial neural network. Methods A shows particle swarm optimization

approach for low sidelobe level (SLL) of the DPM; while Methods B is deep learning approach for the proposed intelligent tracking system. Finally, the advantages and added value of ANN are summarized.

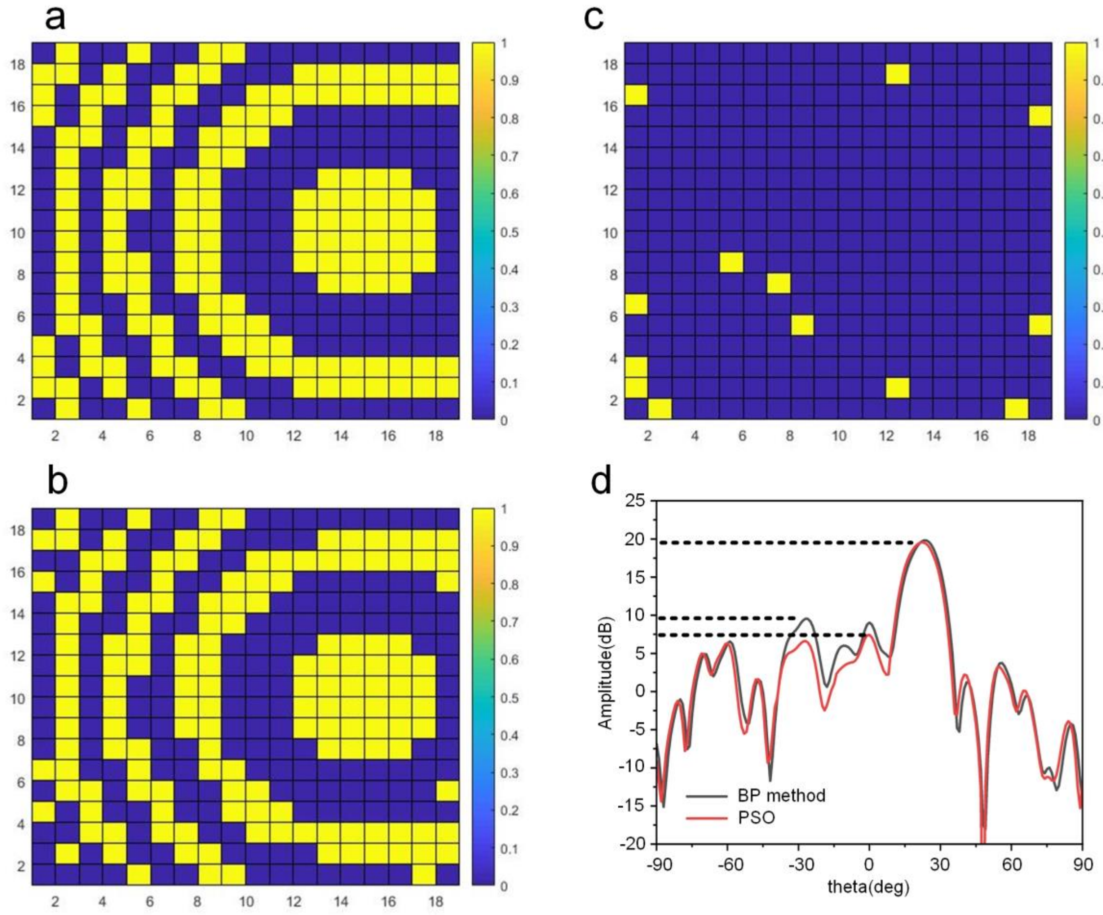

Supplementary Fig. 10 Single-beam coding matrices calculated by (a) back-projection and (b) PSO algorithm. In (c) shows the position where the element is flipped obtained by two approaches and (d) compares the beam pattern on the principle plane generated by these two approaches.

Methods A: Nonlinear Optimization Approach

The calculation of the metasurface coding matrix can be formulated as an optimization problem, to design a given scattering pattern of the metasurface. We generally use random nonlinear optimization algorithms, such as genetic algorithm[8] (GA) or particle swarm optimization[9] (PSO), to approximate the designed optimal coding matrix through iteration. The nonlinear optimization algorithm uses randomness and other characteristics to find the global minimum, which is the coding matrix with the best performance of the metasurface. We use the PSO algorithm to optimize the beam. The following figure shows the coding matrices and simulated

results for realizing a single beam at (θ, φ) = (25°, 0°), the SLL under BP method and PSO optimization is lower than −10.24 dB and −12.13 dB at 5.8 GHz, respectively. When PSO is used for low sidelobe optimization of DPM, the vector dimension is set to 100 and the maximum number of evolutionary iterations is 100.

Methods B: ANN Approach

The deep learning techniques combined with the metasurface can compute the coding matrices for complex beam patterns[10-12]. Regarding the design of reconfigurable metasurfaces at microwave frequencies, some scholars have proposed deep learning-assisted design schemes. According to those application scenarios, the network input of deep learning can be the radiation pattern[12], spectrum information[11,13,14], or the information of incident waves[15]. Since the primary goal of the tracking system is to achieve beam alignment, we take the elevation and azimuth angles of the scattering beam as the input of the network. The input consists of two angels of the scattering beam, as illustrated in Fig. R19. In this intelligent tracking system, the two angles (θ, φ) detected by the RS-camera are directly fed into the network for calculation. No additional operations are required to achieve specific network input forms.

The output of the artificial neural network is the coding matrix of the DPM, which can produce a single beam with low sidelobe that fulfills the realization of the input angle. Fig. R19 shows an example of the output. The output coding matrix consists of binary numbers "1" and "0", which are corresponding to the ON and OFF states of the PIN diodes in the DPM. The reflected beam of the DPM can be manipulated through adjusting the coding pattern.

Here, we give a detailed illustration of the proposed deep learning method of predicting coding matrix from θ and φ. Firstly, the input angels θ and φ are embedded as dense representations. The dimension of embedding is set to be 60. The representations of angles will then be concatenated and input into a 3-layer Multilayer perceptron (MLP). The dimension of each layer is also shown in the Supplementary Fig. 11. We choose Rectified Linear Unit (ReLU) as the final activation function of the MLP and the dimension of the output from the MLP is 1282. The output will then be reshaped as square images with a side length of 128. The generated images based on the angles are inputs of the following ResNet34[16], which tries to predict the coding matrix. For each residual block, we implement batch normalization layer[17]

and ReLU activation. We implement 16 residual blocks and every block have 2 convolution layers. With an additional convolution layer and the last fully-connected layer, we obtain 34 weighted layers in total. The dimension of the output is 18^2 so that it can be reshaped as the same size of the ground truth coding matrix. The activation function for output is sigmoid. We calculate the Binary Cross-Entropy / Log between the changed real patterns and those predicted ones as loss function. The formula of the loss is given as follows.

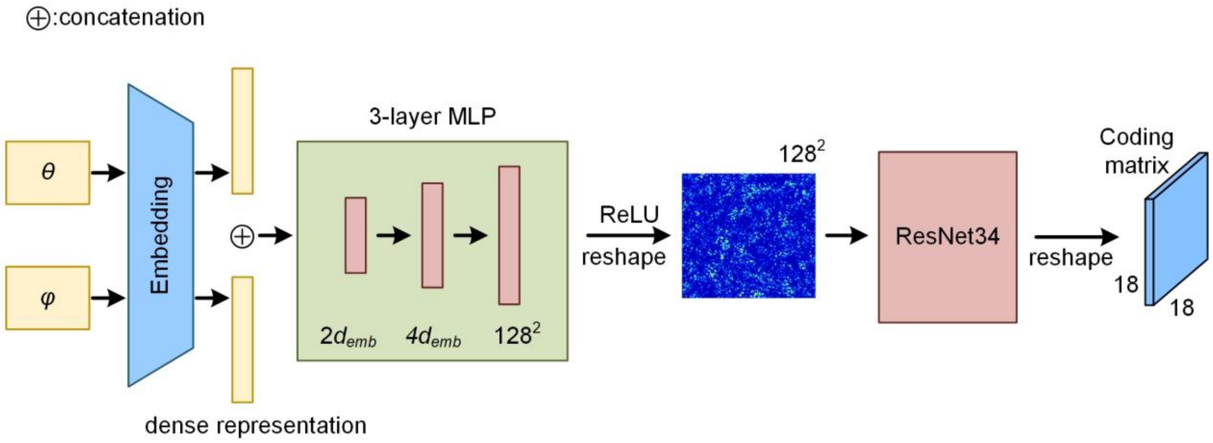

Supplementary Fig. 11 Schematic of proposed artificial neural network (ANN).

$$BCEloss = -\frac{1}{N}\sum_{i=1}^{N}((truth(i) * log(pred(i)) + (1 - truth(i)) * log(1 - pred(i))) \tag{S14}$$

where N is number of coding elements in a pattern, which equals to 18^2 here, *pred* and *truth* indicates the predicted and ground truth coding matrix, respectively. We utilize the Adam optimizer[19] and the learning rate is set to be 2×10^{-5}. When we generate coding matrix with θ and φ in the test set, the positions in the output of ResNet34 with positive values will be encoded as “1” and those with negative values will be regarded as “0”. The prediction accuracy is given by the ratio of the correct elements in the predicted arrays. After proper numerical computation, the corresponding 2D scattering patterns will be obtained from the predicted coding arrays.

In the whole dataset, θ varies from -45° to 45° and φ varies from 0° to 360°. The value range of θ is not -90° to 90° (half-space), because the range of field angle that can be measured by the camera is only about 80° on the θ plane, and when the reflected beam of the DPM exceeds 70°, the main lobe of DPM is difficult to meet, and the phase difference between coding elements is not enough to achieve. Both θ and φ share the same step of variation of 1°, we

randomly optimized low sidelobe codes from more than 12,000 angles, with 80% training, 20% testing. After 200 epochs of training, the prediction precision of coding positions on the test set is 95.32%, which suggests the effectiveness of our proposed model. The evolution of the loss during training is presented in Supplementary Fig. 12. It can be seen that value of loss function declines rapidly as the parameters are optimized.

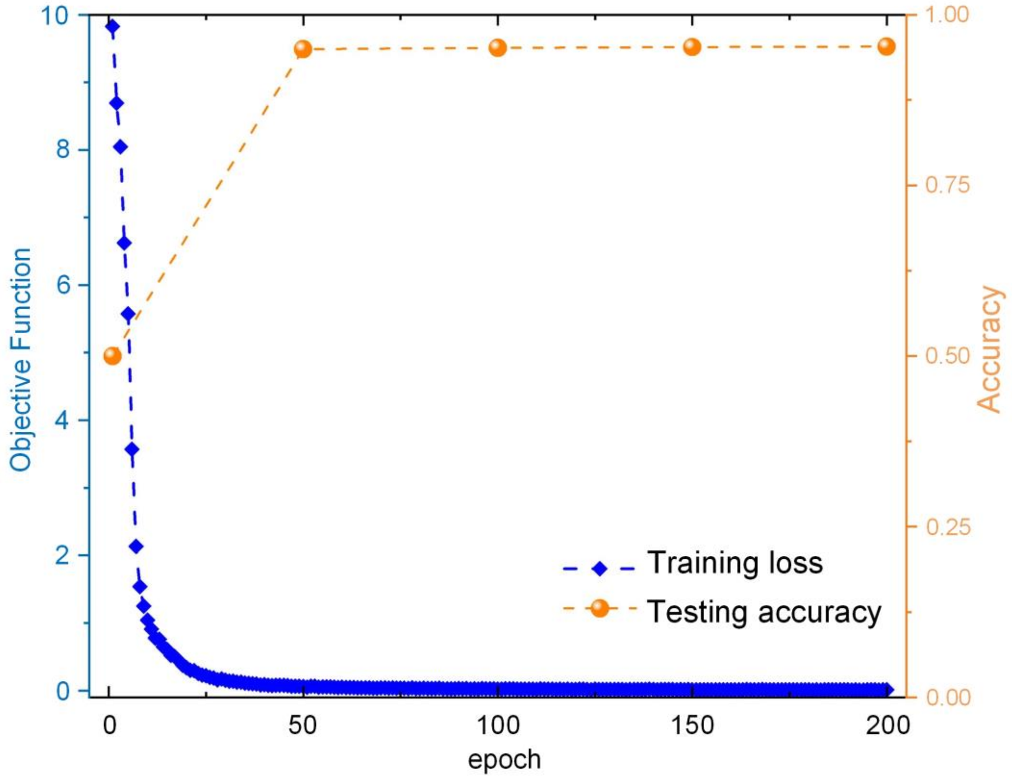

Supplementary Fig. 12 The average loss and accuracy of the training and testing process

Supplementary Note 8. The advantages and usefulness of the proposed ANN approach

The added value of the presented artificial neural networks (ANN) is summarized from three points: a) compared to the theoretical calculation like back-projection[18,19] (BP) the presented algorithm has better beam accuracy and sidelobe performance; b) compared to the nonlinear optimization algorithms like genetic algorithm[8] (GA) and particle swarm optimization (PSO)[9], the presented method raises a much faster speed to obtain the target coding matrix; c) for realistic environment, the presented ANN is able to overcome some interference such as environmental multipath scattering and other interference sources. More details and analysis are provided in the following. Indeed, the controlled condition like an anechoic chamber cannot fully exhibit the advantages of the ANN. Therefore, we supplemented an outdoor-field

experiment (please refer to the Supplementary Note 15 for details) to demonstrate that the presented ANN can work in the complex environment. The measurement results proved that the ANN has the capabilities to solve the interference and guarantee the high signal-noise ratio (SNR) (please refer to Supplementary Note 16 for details).

In summary, our ANN method has the following advantages:

1. A higher speed. Respond in real time when coding the programmable metasurface. For a more intuitive comparison, we examine the computing time of the above three schemes to generate one coding matrix. It takes the back-projection approach 0.003 seconds, it takes the PSO algorithm about 25 seconds for a low sidelobe case in this paper. The ANN approach it takes an average of 0.002 seconds. The computation platform is Intel Core i7-9750H CPU @2.60GHz and accelerated by one Nvidia GTX 1650 GPU. Platforms are different, and different methods take different amounts of time, and while that's not a fair comparison, it still indicates that the ANN approach can provide not only accurate but also real-time responses when coding the programmable metasurface.
2. Performs well with complex scattering problems. For complex beam requirements, global results can be accurately output through relatively large datasets.
3. Anti-interference. The proposed ANN can work normally in the outdoor environment, showing good anti-interference characteristics. The digital programmable metasurface (DPM) modulates incident waves from the feeding horn and creates flexible and controllable radiating beams in dual polarization. The proposed ANN can be flexibly deployed in different directions of waves and specific usage scenarios. Please refer to Supplementary Note 15 and 17 for details. Compared with traditional computing methods, artificial neural networks can solve complex scattering field in real time. It also has the characteristics of considering the external EM environment and has stronger anti-interference ability. Assuming that the current EM environment and noise are stable, we can collect the EM environment information of the system in advance to customize an adaptive and easily deployed network in the current environment. In the case of non-interference scenarios, the original parameters can be fine-tuned, without training from the beginning, and quickly deployed to various scenarios.
4. Although ANN usually requires a large time overhead in the training process, a well-trained

ANN model has significant advantages in practical applications.

To specifically exhibit the above advantages, we compare the presented ANN with other methods in terms of speed, side-lobe and anti-interference. Table.S2 lists the speed and low-sidelobe performance after executions of BP, PSO optimization and the proposed ANN. The speed calculation of the three methods is based on the design of the 18×18 digital programmable metasurface (DPM). The low-sidelobe solution is not applicable (N/A) using the BP method, but is available through the PSO optimization and the proposed ANN method. For anti-interference capability, first of all we comprehensively consider low side-lobes8-10 which are important to communication SNR. In addition, the ANN is designed to use actual measurements as a training set to obtain more practical results when compared with the nonlinear optimization methods. The adjacent elements of the DPM are not independent, but a whole formed by interaction and coupling that are difficult to be optimized. Also, the higher order scattered waves are difficult to be predicted by the optimization methods. Therefore, from data collection to algorithm modeling to experimental demonstration, globally designed algorithms are required. In view of these, adopting the measured results as a training set to obtain more accurate output, ANN provides added value for complex beam requirements and anti-interference problems[13].

Supplementary Table 2 lists the speed and low-sidelobe performance after executions of BP, PSO optimization and the proposed ANN.

Supplementary Table 2. The results derived from BP, PSO optimization, and the proposed ANN

Method	Speed[7]	Low side lobe	Anti-interference[6,14]
Back-Projection	≤5 ms	N/A	Low
Nonlinear Optimization	≥ 20 s	Available	Adequate
ANN	≤5 ms	Available	High

Supplementary Note 9. Design of the receiver patch antenna

Supplementary Fig. 13(a-b) shows the basic structure of the proposed patch antenna that serves as the receiver. This U-shape patch antenna has a total size of 80×80 mm^2. The commercial dielectric F4B with a relative permittivity of 2.65, a thickness of 1.5 mm, and a loss tangent of

0.003 is adopted in this design. The dimensions of the patch are listed in Supplementary Table 3. Supplementary Fig. 13(c) gives the simulated and measured reflection coefficient (S11) for the patch antenna, showing that it works around 5.77 GHz. The simulated gain is also given in the inset, proving that the antenna has a very good radiation performance.

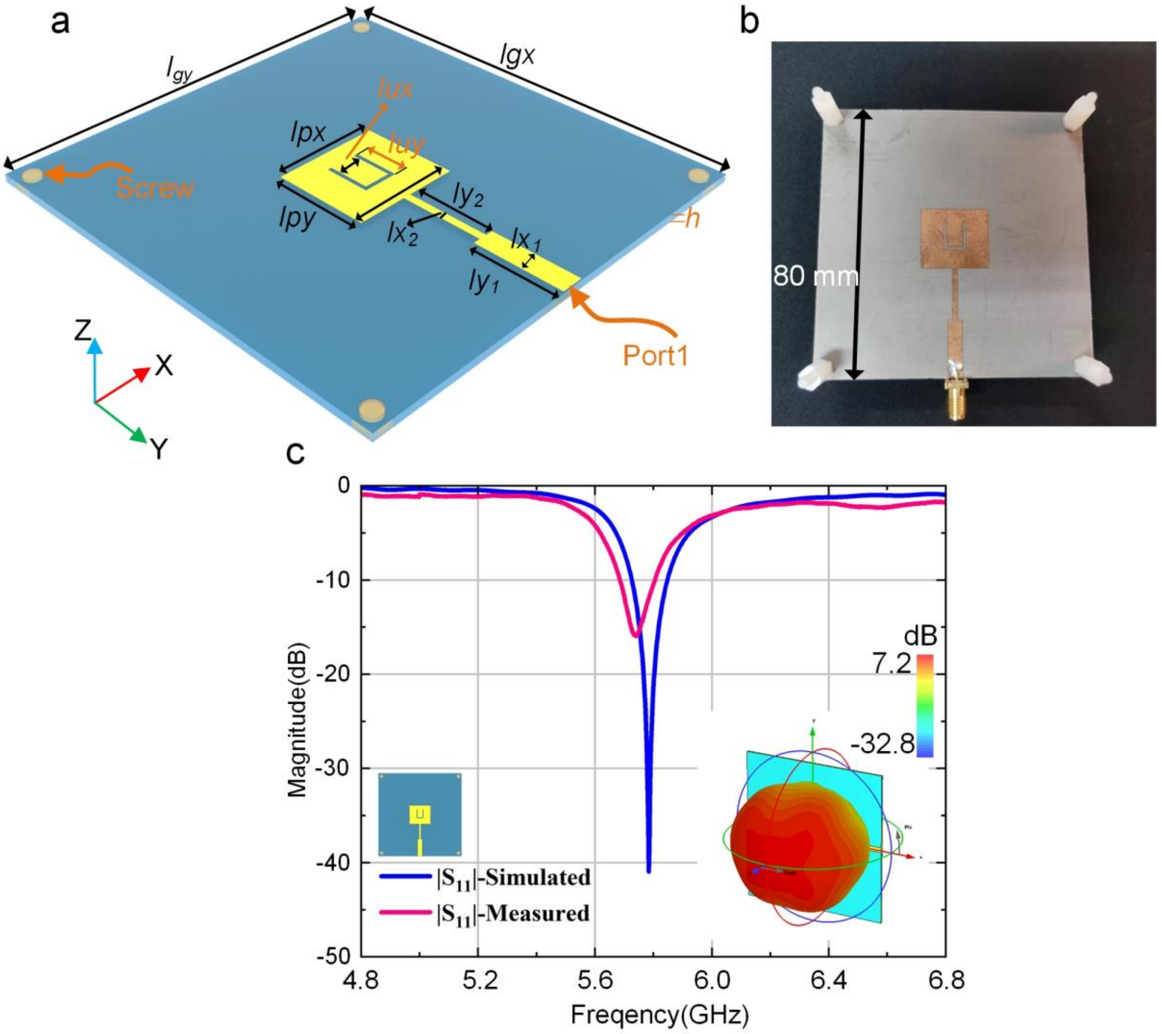

Supplementary Fig. 13 The receiver patch antenna. (a) Perspective view of the antenna. (b) Photograph of the fabricated antenna. (c) The gain and reflection coefficient (S11) of the patch antenna.

Supplementary Table 3. Dimensions of the patch antenna.

Parameters	lx_1	ly_1	lx_2	ly_2	lux	luy	lpx	lpy	lgx	lgy	h
Dimension (mm)	4.1	15.95	1.4	15	5	8.4	20	17.6	80	80	1.5

Supplementary Note 10. Description of switching speed of system

We measured the switching speed of FPGA and the switching speed of the whole system, and

got real-time data. As shown in Supplementary Fig. 14(a), we use serial port tools and software to send sequences to FPGA at regular intervals. The pins of the FPGA are connected with the logic analyzer, and another computer is used to monitor the results of logic analyzer. We find that the FPGA can complete the voltage change on the pins with sequences at intervals of about 30ms, and the results are shown in Supplementary Fig. 14(b). Similarly, we switched our system to the first serial port tool in the experiment. Through the previous analysis, we can track the moving target at a relatively fast speed, and each identification will send data. We can control the time interval of sending data. In the experiment, we set it to 0.2s, and the time interval between two samples obtained by testing with logic analyzer is about 0.2s, which is in line with the time domain change of the system.

We can also choose to send a voltage sequence to the FPGA with each sampling as shown in Supplementary Fig. 14(e), which can increase the speed. Because the moving speed of the object is not very fast and in order to save energy consumption, we choose to send a voltage sequence to the FPGA every three frames detected. According to the situation of the existing system, if the detection of each frame is sent to the FPGA, the speed is about 79.22ms. In addition, cameras with faster frame rates and computers with more processing speed can help our system to respond faster.

Supplementary Note 11. Collection and production of data sets. The performance of target tracking algorithms and experimental results in the case of multi-object tracking (MOT), and when the target is might be temporarily blocked.

First, we describe the collection and production of data sets. In this experiment, the RS-camera was used to sample the tracked target (the model car with a portable RF signal detector attached on it here). The RS-camera takes pictures of the captured samples 3 times per second, and sends them as pictures for saving. We denote that the number of samples per second can be flexibly set, and in this work we chose the rate of 3 samples per second to ensure that there is no excessive repetition of samples.

During the sampling process, the moving target is captured at different positions of the field of view with different postures, so as to ensure that as many image samples of the target are collected as possible. Additional manual screening may help to remove some data with too

much interference, leaving images with typical characteristics. These data are annotated through the tool “labelImg”, and the annotation information is saved as an xml file, which becomes the collection and processing of datasets, as shown in the Supplementary Fig. 15(a) below. The sampling frequency and the label given to the target can be modified as required.

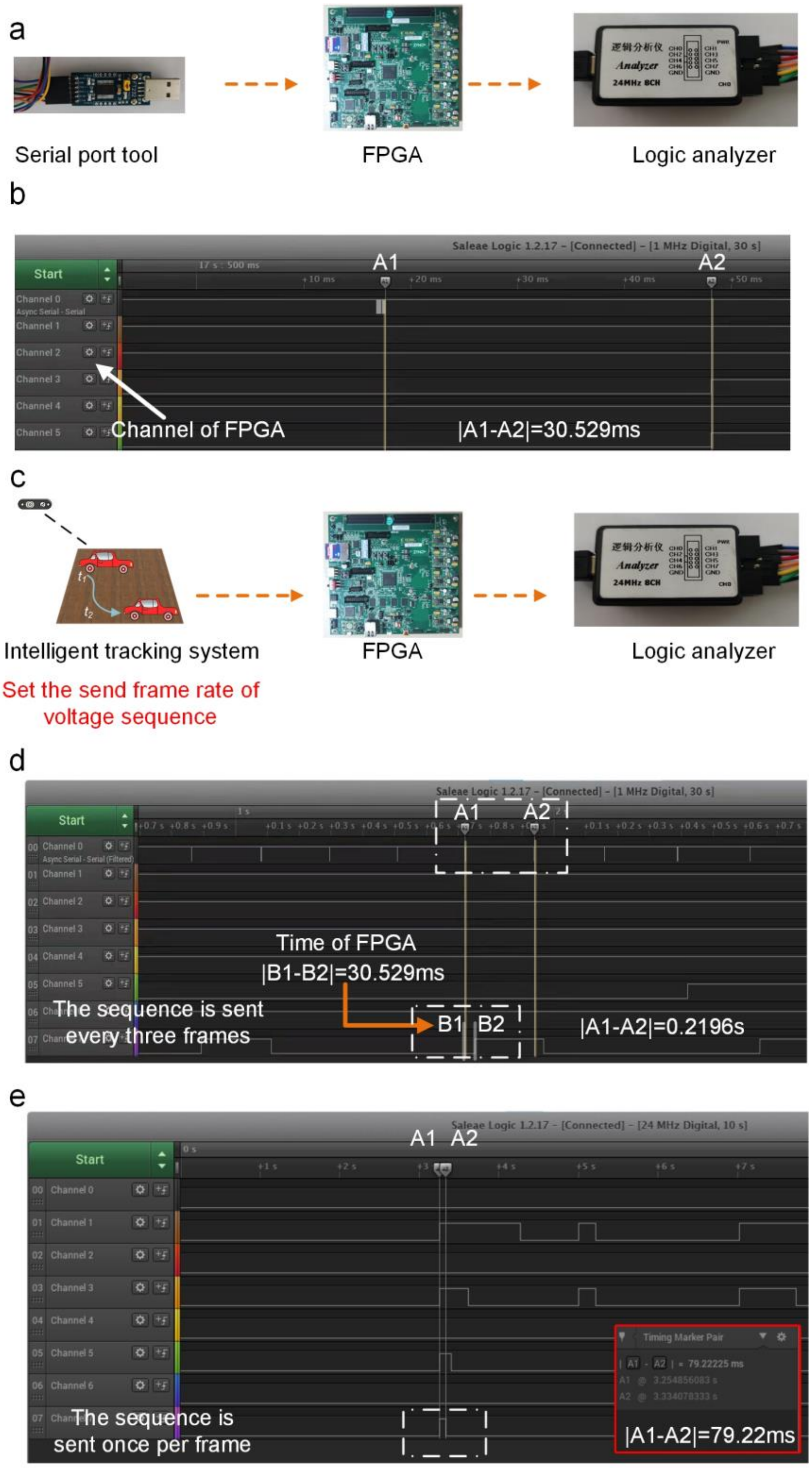

Supplementary Fig. 14 Experimental test of switching speed and actual sampling results at different send frame rates. (a, b) Experimental setup and results of FPGA response speed. Experimental setup of the

response speed of the intelligent track system (as given in figure (c)). Results of the response speed of the intelligent track system, when the coding sequence is sent every three frames (as indicated in (d)) and once per frame (as indicated in (e)).

Next, the performance of target tracking algorithms and experimental results of multiple targets and target occlusion is described below.

Similar target interference and target occlusion are two key problems for MOT[20]. To solve the problem of similar target interference, multiple targets in the visual field are numbered and the corresponding numbers of them in the video stream are guaranteed to remain unchanged. This task can be completed by matching between the results of object detection in the preceding frame and the following one, where the two detected boxes with high similarity are considered to be the same target and assigned the same number. In the deep simple online and realtime tracking (SORT) algorithm proposed in literature[21], the similarity consists of the appearance feature similarity, which is the cosine distance between the features extracted by the convolutional neural network, and the spatial information similarity, which is the mahalanobis distance between the two detection boxes. The cost matrix between the tracker and the current detection box of frame is obtained by calculating the similarity, and then the Hungarian algorithm is used to find the optimal match. For the problem of target occlusion, the deep SORT algorithm introduces a Kalman filter to solve the problem of transient target occlusion and the problem of missing individual frame detection. The algorithm initializes a Kalman filter for each tracker, and after the optimal match is obtained by the Hungarian algorithm, there are three kind state for trackers and detection boxes at this moment: successfully matched trackers and detection boxes, unmatched trackers, and unmatched detection boxes. (1) for the successfully matched tracker and detection boxes, the detection box is updated as a new observation to the Kalman filter; (2) for the unmatched tracker, the predicted value of the Kalman filter is used as the target state of the frame; (3) for the unmatched detection box, it is initialized as a new tracker and assigned a new number. In addition, the algorithm sets the maximum survival time of the Kalman filter, i.e. when no observation is obtained for n consecutive frames of the tracker, the target is considered lost and the information of that number is cleared.

In order to verify the performance of the proposed system when multiple similar targets and target occlusion are included, we conducted three groups of experiments. The first group is

in the scenario when multiple similar targets are included, and the second one group is when the target is temporarily occluded. We demonstrate that the algorithm runs stable enough to complete detection and tracking tasks in these two scenarios. And the third group is the verification of energy reception in the case of multiple similar targets.

In the first group of experiments, we numbered multiple targets, and then manually entered the number of the tracked target. Location of the tracked target is presented in black. As shown in Supplementary Fig. 15 (b), location of car No. 1 was firstly tracked as the cars moving from right to left, and then the tracked target was switched to No. 3 (see Supplementary Fig. 15 (c)), and then to No. 2 (see Supplementary Fig. 15 (d)). Supplementary Fig. 15 (e) shows the information processed for all targets. From top to bottom, the confidence degree of car recognition, the location information of each car, and the number of the tracked car were respectively recorded. From the experimental results, it is verified that each car can be switched at any time, and the algorithm runs stable enough to complete the detection and tracking tasks with multiple similar targets.

In the second group of experiments, the target was temporarily occluded by the blue foam of shelter. Supplementary Fig. 15(f-i) respectively shows the recognition of the target before occlusion, the recognition when the target is occluded, and the reappearance of the target after occlusion. Supplementary Fig. 15(i) displays the information of the object detection when the target was occluded. From the experimental results, it can be seen that the tracking box of the car keeps moving even when the car is occluded by the shelter. It is judged and predicted by the situation of previous frame rates. In view of this, we conclude that the algorithm runs stably and can complete the tracking task when the target is occluded.

In the third experiment, we rely on a prototype of the DPM to demonstrate that the tracked target can effectively receive energy from the source when multiple similar targets are involved. The experimental setup is the same as the one in the section of “Moving target detection and identification” of the original submission. The DPM as the transmitter was fed with a linearly polarized horn antenna connected to a vector network analyzer (VNA), and a patch antenna designed at 5.77 GHz as the receiver was located in the middle of the moving path of the cars. The VNA tested the energy received by the patch antenna (in term of S21) to verify the multi-object detection.

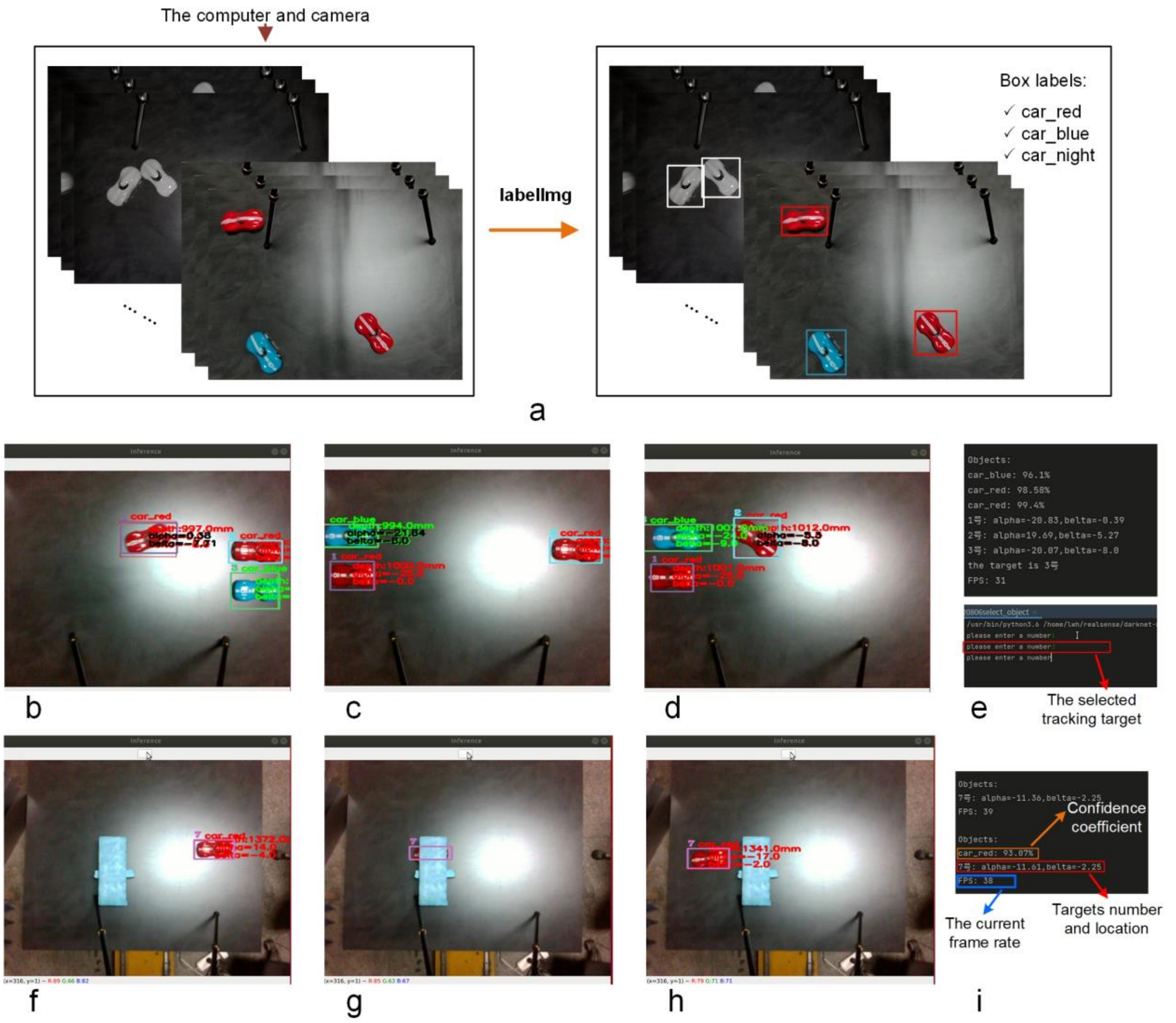

Supplementary Fig. 15 (a) Flow chart of dataset processing using labelImg. Performance of the object detection algorithm in scenarios where (b-e) multiple similar targets and (f-i) temporary target occlusion happened. Here, two red and one blue cars of the same model were used as multiple similar targets. (b-d) are three cars moving from the right side to the left side of the scene. In the movement, the three targets are individually numbered and the position information of the tracked one is presented in black. (f-h) A moving target is temporarily blocked by the blue foam of shelter, but the tracking box can predict the target (as indicated in (g)) and thus complete the tracking task (as given in figure (i)). (see Supplementary Movie 2 for Referee_1- Comment_3 for details).

Figures S16(a-d) record four typical states in the movement of the cars. The blue car (numbered 1) ran first from right to left, but was not selected as the tracked target, and hence the energy received by the patch remained basically unchanged and low, as is indicated by the S21 curve in (a). Then the red car (numbered 3), which was chosen as the tracked target, started to run. As it moved from right to left, the S21 curve increased in (c) when it was near the patch

and decreased in (d) when it left the patch. The other red car (numbered 2) was at rest as the reference of multiple targets. Supplementary Fig. 16 (e) is the flow chart of the movements in the experiment and (f) shows the S21 measured in VNA over time. Clearly, this system can effectively fulfill communication to the tracked target even when similar targets exist nearby.

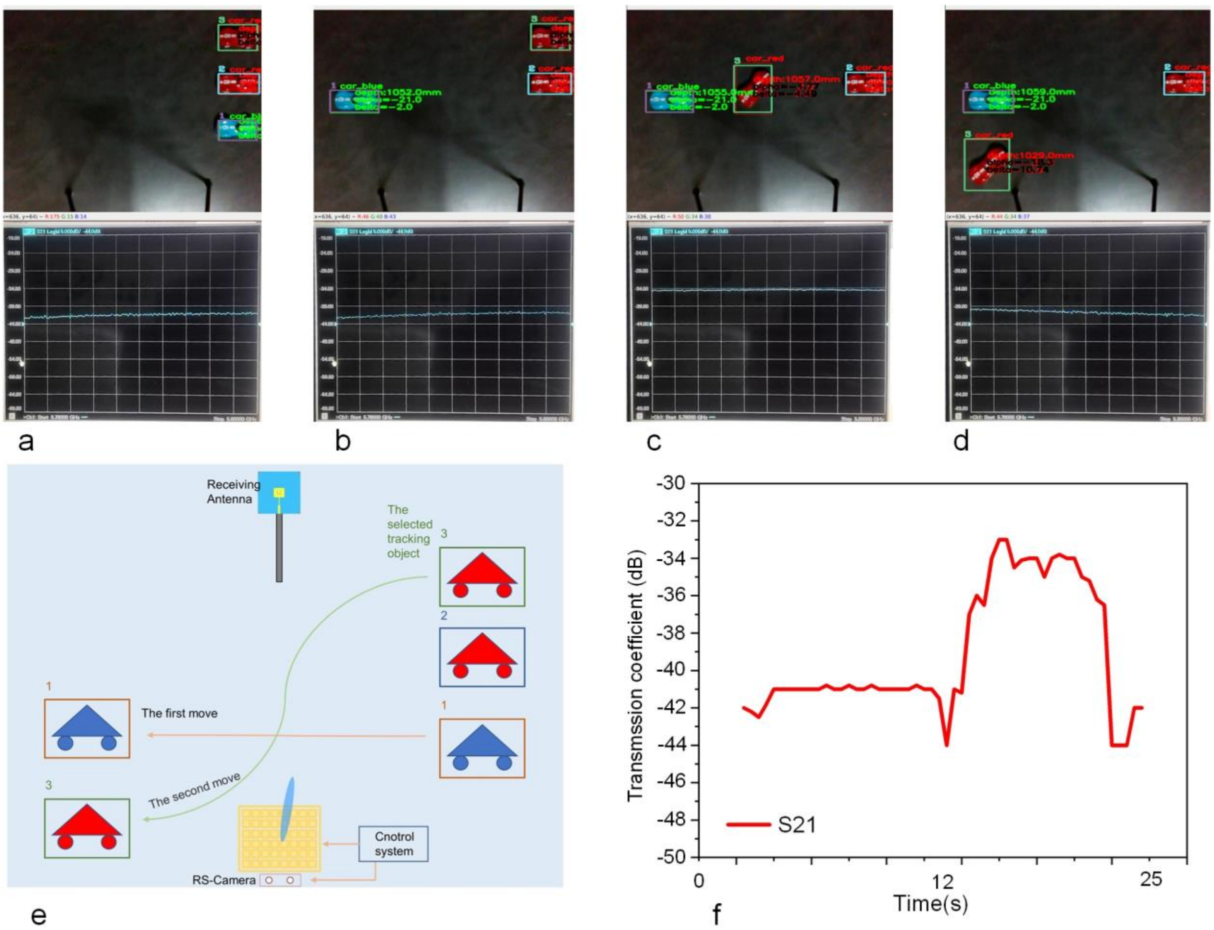

Supplementary Fig. 16 Experiments to verify the tracking scheme when multiple similar targets are involved. Two red cars and one blue car of the same model were used as the multiple similar targets. We put the patch receiver in the middle of the moving path of the cars, and the VNA tested the received energy in term of S21 to verify the multi-object detection. (a-d) record four typical states in the movement of the cars. The blue car (numbered 1) ran first from right to left, but was not selected as the tracked target, and the energy received by the patch remained basically unchanged and low, as is indicated by the S21 curve in (a). Then the red car (numbered 3), which was chosen as the tracked target, started to run. As it moved from right to left, the S21 curve increased in (c) when it was near the patch and decreased again in (d) when it left the patch. The other red car (numbered 2) was at rest as the reference of multiple targets. (f) shows the S21 result of VNA over time. (e) is the flow chart of the movements in the experiment. (see Supplementary Movie 3 for Referee_1-Comment_3_exp_vna for details).

Supplementary Note 12. Description of experiments with multiple different classes of targets

In this experiment, we demonstrate that the system can identify multiple targets and intelligently switch the target to be tracked. YOLOv4-tiny target detection algorithm can classify multiple targets in the field of vision at the same time, and decide the categories to which the targets belong. By judging the category, the position information of the specified target is extracted, and the beam is controlled to point to the specified target. Here, the first model car is located at the start point of the moving path, whilst the second car is located in the middle of the path, close to the fixed receiving antenna. As the first car moves towards the middle of the path, the system recognizes two cars but only returns the position information of the first car. Therefore, the directive beam of DPM tracks the first car in the first half path, and the power received by the receiving antenna gradually increases as the first car approaches. Beyond the midpoint of the moving path, the first car stops and the second car starts to move. Since multi-target recognition can switch between different targets and return the required target information, the beam automatically switches to track the second car in the second half path. Consequently, as the second car moves towards the end of the path, the energy received by the receiving antenna gradually decreases (see Supplementary Movie 4 for details).

Supplementary Note 13. Performance of target tracking algorithms and experimental results in the case of limited ambient light

In computer vision (CV) tasks based on image vision, different light intensities are often encountered, which affects the contrast of images and consequently the final result of CV tasks. In many realistic scenarios, due to the lack of robustness of computer vision, multi-sensor fusion should be paid more attention in future research and practical applications. Visual scene understanding in complex scenarios is a problem that must be solved through combining computer vision tasks with applications (such as the unmanned driving and robots). Therefore, in complex environment, it is necessary to optimize and upgrade the hardware and software of a variety of new imaging research technologies. For example, the infrared thermal imager, which works in dark environment, not only has a wide application value in the industrial field, but also have an important application in epidemic prevention and public safety. Hardware

synchronization and software integration of infrared image and visible image will make it easier to solve the problem of object detection in limited ambient light.

In order to answer this question in a more straightforward way, we use night vision (NV-) infrared-cut camera as an aid to solve the detection task under the condition of insufficient light and darkness. In the experiment, a digital photometer was used to test the light intensity, and the digital photometer was placed in the lower right corner of the test scene. In order to control the reflection of light on the floor of the test platform, please allow us to place absorbing cotton on the platform. First of all, using the existing experimental equipment in the manuscript, we carried out eight experiments to test the object detection algorithm under different light intensities. Among them, RS-Camera was used in the first six experiments, and NV-Camera was used in the last two experiments. All the experiments were conducted in the room, as shown in Supplementary Fig. 17(a). The first experiment tested a scene with a natural light intensity of 290.0 lux, and the second to the sixth experiments used an adjustable light source, as shown in Supplementary Fig. 17(b-f). From the results of RS-Camera, we can see that when the light intensity is greater than or equal to 5.9 lux, the object detection algorithm can complete the target tracking task. It was observed in experiment that when the light intensity is around 2.7 lux, the performance of the object detection algorithm of RS-camera is less effective, and it is difficult to meet the stable detection. Supplementary Fig. 17(f) shows that when the light intensity is lower (2.7 lux), the detection cannot be completed, and in some areas we cannot detect the target. Therefore, it can be concluded that when the light intensity is less than 5.9 lux or it is completely black, the real-time tracking function cannot be effectively realized.

Next, we changed the responsibility for obtaining the target position information to the NV-Camera, and carried out the last two experiments, as shown in Supplementary Fig. 17(g-h). The light intensity from left to right was 5.9 lux and 0.0 lux, respectively, and 0.0 lux represented completely dark. From the experimental results, it is observed that the NV-Camera can complete the detection of moving targets with an average confidence coefficient of over 95% under very low light intensity or even in completely dark. We denote that the NV-Camera and RS-Camera are both used as vision sensors, in which an image processing device reads image data from the camera and performs vision algorithm processing. The main difference between them is that NV-Camera can obtain the infrared image of the target in a dark

environment, but without the depth of the target. In other words, the NV-Camera can only obtain the elevation and azimuth angles of the object in the camera's coordinate system. NV-Camera can automatically switch to night vision when the illumination is low or completely dark. In contrast, the RS-Camera can get the depth of the target in a well-lit environment so as to obtain the 3D coordinates of the target, but is not able to obtain effective images in a dark environment. For our system, NV-Camera is used in conjunction with the RS-Camera. When the illumination is below 5.9 lux, it is switched to NV-Camera to complete the system operation. Under good lighting conditions, RS-Camera is used to obtain the detailed position of the object. (see Supplementary Response Movie 5 for details).

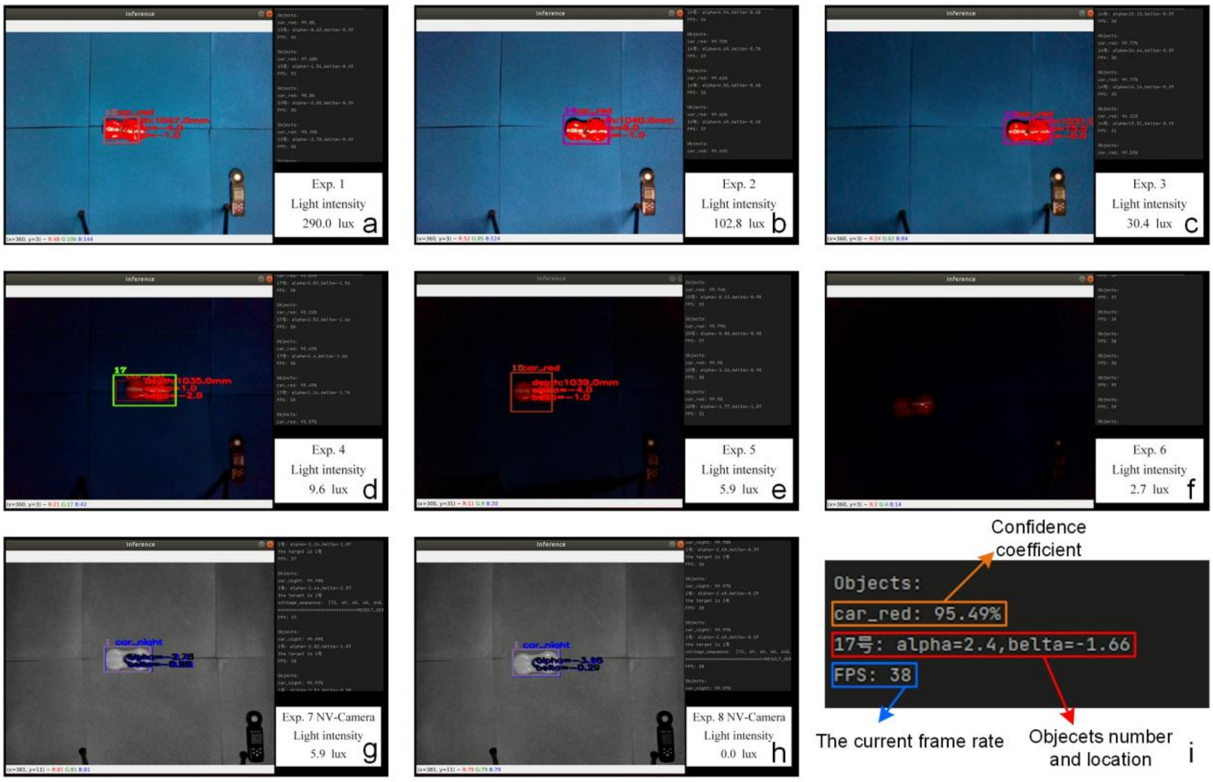

Supplementary Fig. 17 Performance of the object detection algorithm under different light intensities. Here, (a-h), indoor, we used adjustable light source, and carried out experiments with different light intensity. (First and second rows, RS-camera, left to right: 290.0 lux, 102.8 lux, 30.4 lux, 9.6 lux, 5.9 lux and 2.7 lux. The third row, NV-Camera, left to right: 5.9 lux and 0.0 lux). Blue foam was put on the floor to control the reflection of the floor. When the light intensity is low (5.9 lux) or completely dark (0 lux), the NV-Camera helps to obtain the infrared image of the target. The NV-Camera cannot get the depth of the target, so only the elevation and azimuth angles of the object are given in (i).

Supplementary Note 14. Description of working mechanism of the detector AD8317

The AD8317 is a 6-stage demodulating logarithmic amplifier, specifically designed for the use

in RF measurement and power control applications at frequencies up to 10 GHz[22]. A block diagram is shown in Supplementary Fig. 18(a). Using precision biasing, the gain is stabilized over temperature and supply variations. The overall dc gain is high, due to the cascaded nature of the gain stages. An offset compensation loop is included to correct for offsets within the cascaded cells. At the output of each of the gain stages, a square-law detector cell is used to rectify the signal. The RF signal voltages are converted to a fluctuating differential current having an average value that increases with signal level. Along with the six gain stages and detector cells, an additional detector is included at the input of the AD8317, providing 50 dB dynamic range in total.

The output voltage vs. input signal voltage of the AD8317 is linear-in-dB over a multidecade range. The equation for this function is

$$V_{OUT} = X \times V_{SLOPE/DEC} \times \log_{10}(V_{IN}/V_{INTERCEPT})$$
$$= X \times V_{SLOPE/dB} \times 20 \times \log_{10}(V_{IN}/V_{INTERCEPT}) \quad \text{(S15)}$$

where:

X is the feedback factor in $V_{SET} = V_{OUT}/X$.

$V_{SLOPE/DEC}$ is nominally −440 mV/decade, or −22 mV/dB.

$V_{INTERCEPT}$ is the *x*-axis intercept of the linear-in-dB portion of the V_{OUT} vs. P_{IN} curve (see Supplementary Fig. 18(c)). $V_{SLOPE/DEC}$ represents the volts/decade. A decade corresponds to 20 dB; $V_{SLOPE/DEC}/20 = V_{SLOPE/dB}$ represents the slope in volts/dB.

These parameters are very stable against supply and temperature variations. The input dynamic range is typically 55 dB (referenced to 50 Ω) with less than ±3 dB error. The AD8317 has 6 ns/10 ns response time (fall time/rise time) that enables RF burst detection to a pulse rate of beyond 50 MHz. The device provides unprecedented logarithmic intercept stability vs. ambient temperature conditions. A supply of 3.0 V to 5.5 V is required to power the device. Supplementary Fig. 18 (c) is results of V_{OUT} and input amplitude P_{IN} at 5.8 GHz based on actual measurement, and we take the actual result as reference.

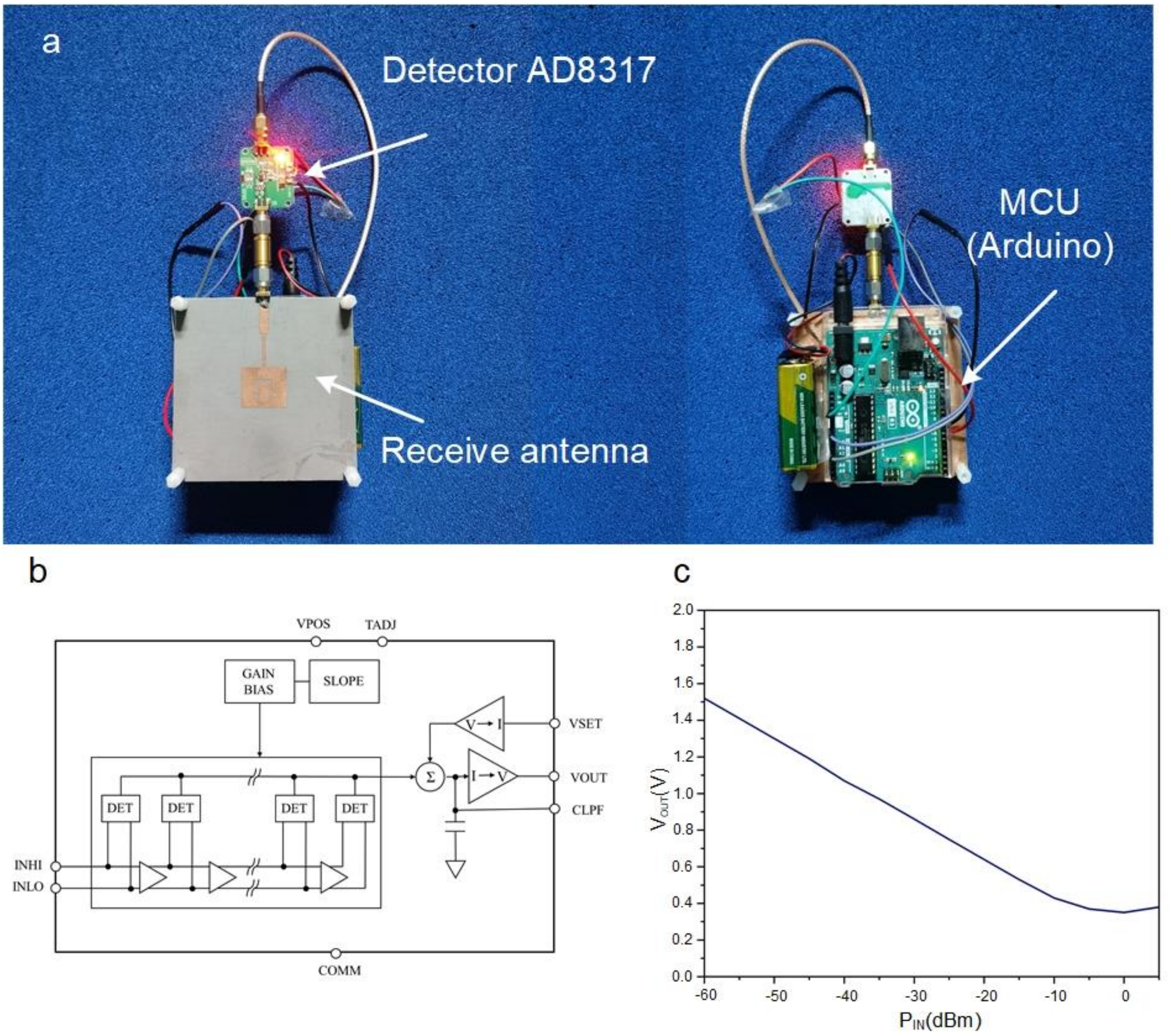

Supplementary Fig. 18 Working mechanism of the detector AD8317. (a)The detector AD8317 is connected to the patch antenna from the front. On the back of detector, the battery supplies powers to MCU, and the power port on MCU supplies powers to AD8317. (b) Functional block diagram of AD8317. (c) V_{OUT} vs. input amplitude (P_{IN}) at 5.8 GHz, the measured results.

Supplementary Note 15. Detailed of outdoor test results

In this experiment, we carried out experiments in an outdoor environment to conduct the real-time tracking scheme in dual polarization. The testing sites were chosen at the campus of Southeast University (SEU). Supplementary Fig. 19 shows the outdoor environment and the experimental setup, including the feeding horn, the DPM, the RF signal detector on the model car, the signal generator, and the FPGA module and control system. The RF signal detector was always placed on the moving car to detect the energy of the EM wave in real time. It consisted of a receiving patch antenna, a battery, a detector AD8317, and a microcontroller unit (MCU).

Detector AD8317 was adopted to accurately measure the RF signal power in the band of 1MHz-10GHz and convert the RF input signal to the corresponding dB scale with accurate logarithmic consistency. The input of the detector AD8317 was connected to the receiving patch antenna, and the output of it was connected to MCU for monitoring and processing in real time. In this way, the portable detector without additional voltage source was realized.

We executed RF signal detection under dual-polarization wireless transmission channel in this experiment. Polarization state of the feeding horn and the receiving patch antenna were changed to perform different polarizations. In this realistic outdoor environment, the RS-camera can still correctly capture the moving target, which is the detector-loaded car here, in the identification process. Four curves in Supplementary Fig. 19(d, e) respectively plot the voltage values obtained by the detector and the corresponding dB calibration values under dual-polarization. When the detector and the car move together, the received energy is relatively stable with a high value. This number is well aligned with the power gain observed in the indoor test.

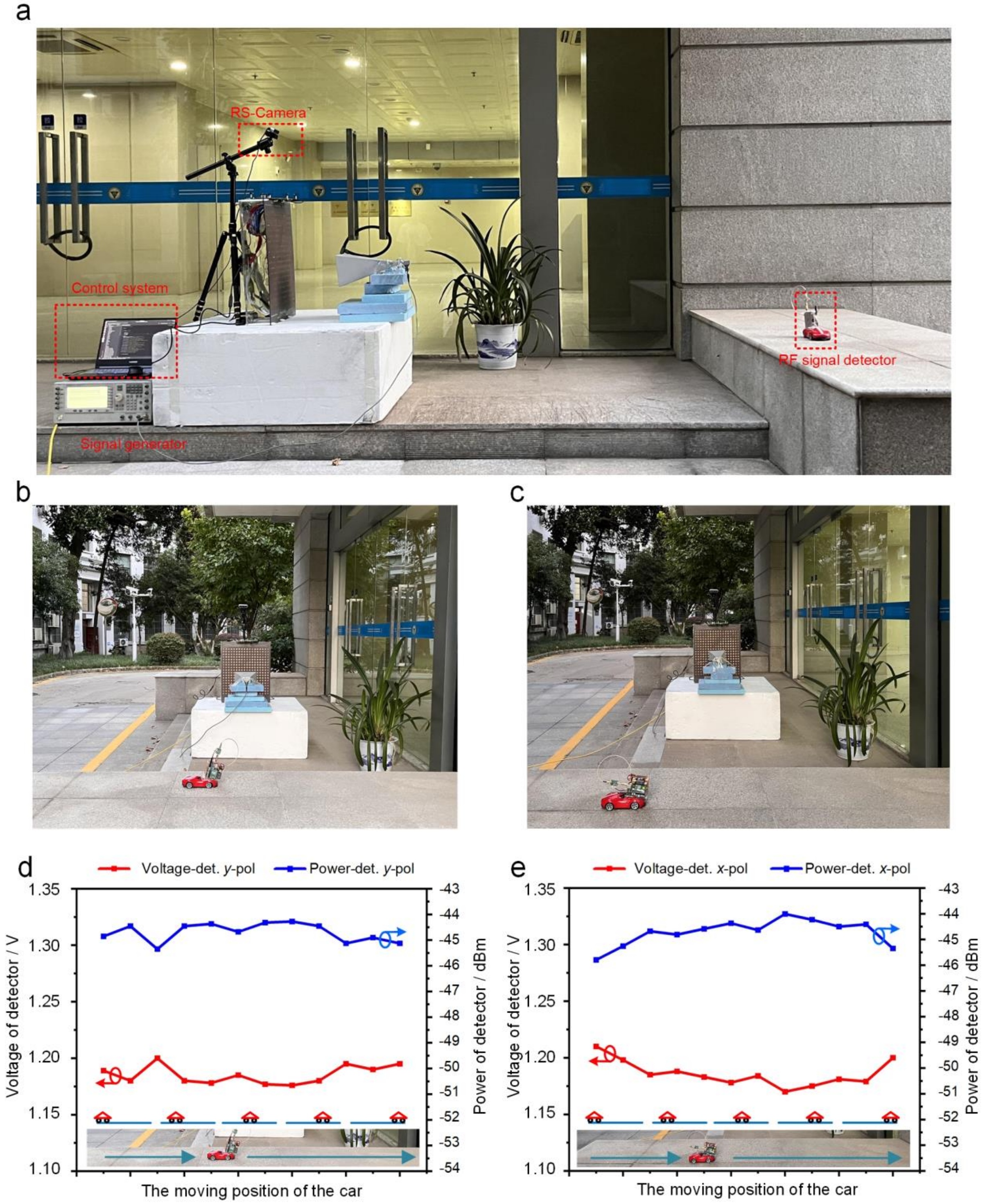

Supplementary Fig. 19. Outdoor experiment of the intelligent tracking system. (a) Setup of the RF signal detection experiment. (b) *y*-polarized wireless transmission channel, (c) *x*-polarized wireless transmission channel. For scenarios with different polarizations, we changed the feeding polarization of the DPM and the orientation of the RF signal detection module attached to the car. RF signal changes under (d) *y*-polarization and (e) *x*-polarization when the detector moves with the car. The horizontal ordinate is the moving path of the car from the beginning to the end.

Supplementary Note 16. Experiments of BER test

Bit error rate (BER) is an important index in wireless communication, which can express the accuracy of data transmission. To test the BER value, a realistic wireless communication system was built to perform experiments of direct data transmission in an indoor scenario, as shown in Supplementary Fig. 20 (a) and 21 (a). The experimental setup is applied to test the BER performance of the wireless communication system and the compatibility of the wireless communication system with different modulation modes. The vector signal transceiver (VST, PXIe-5841, National Instruments Corp.) in the figure is used for the BER measurement. The instrument has the function of setting different operating frequencies, modulation modes and bit transmission rates. We take the DPM fed by a linearly polarized horn antenna as the transmitting terminal, and the receiving antenna is fixed somewhere on the moving path of the target (in Supplementary Fig. 20(a)) or on the moving model car (in Supplementary Fig. 21(a)). The transmitter and the receiver are kept the same height from the ground. The transmitter is connected to the signal output port of the VST, and the receiver is connected to the signal input port of the VST.

The commonly used modulation modes include quadrature phase shift keying (QPSK), quadrature amplitude modulation (QAM), and so on. In this BER experiment, the sinusoidal carrier of QPSK signal has four possible discrete phase states, and each carrier phase carries two binary symbols. Bit rate, also known as "binary bit rate", is used to describe the transmission rate of wireless communication systems. It represents the number of bits transmitted per unit time (commonly written as bps). Generally speaking, by changing the parameter settings of the VST, the signal transmission test with different bit rates can be realized.

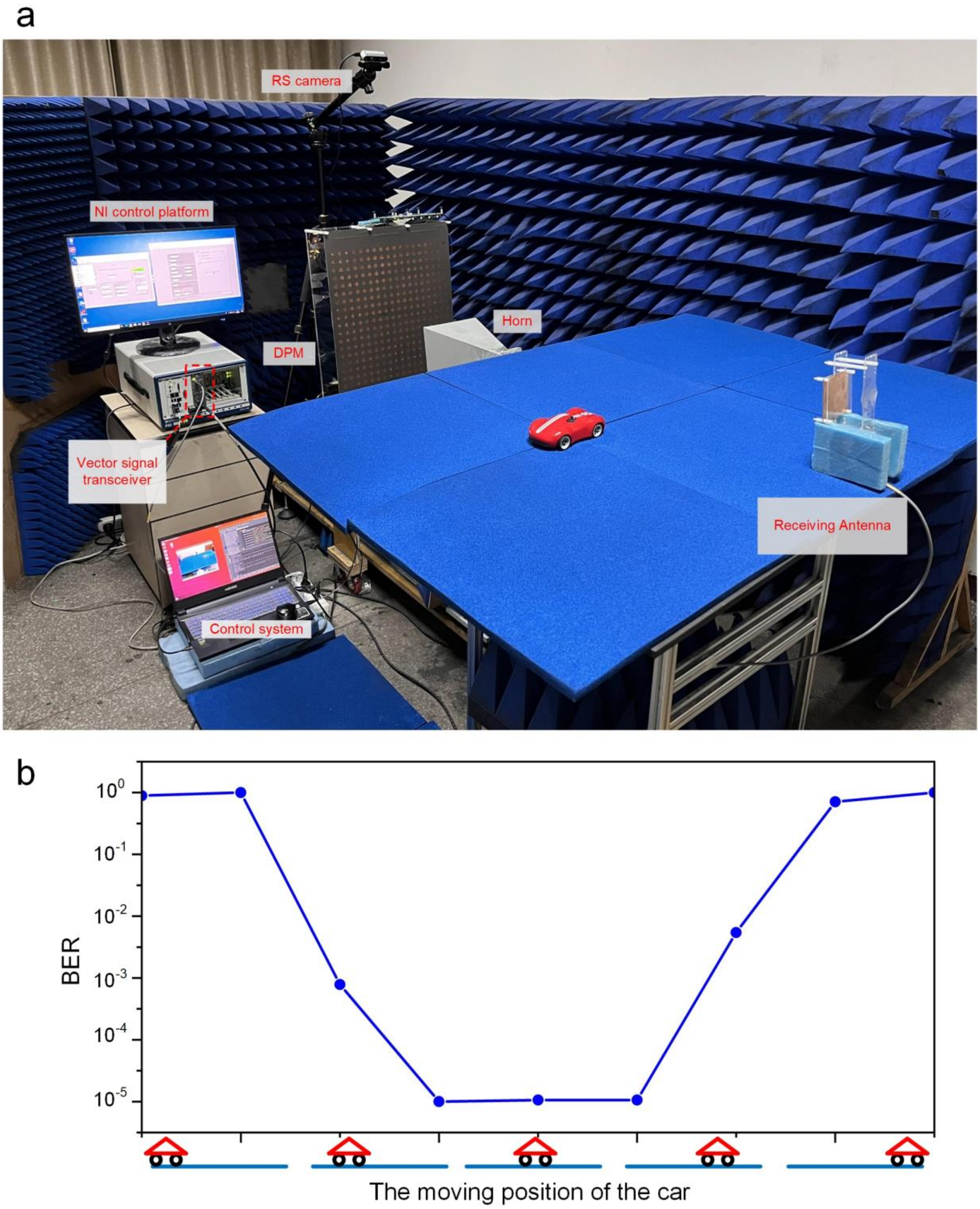

Supplementary Fig. 20 (a) Experimental setup of BER testing. Two colorful pictures were transmitted from the transmitter (the horn) to the standing receiver (the receiving antenna), with an information transmission rate of 170 Mbps at the frequency of 5.8 GHz. (b) The measured value of BER.

In the two experiments of BER test, we set the modulation mode to QPSK and the transmission rate to 170 Mbps, read the data in the display panel of the VST and record the results. In the first experiment, it is observed in Supplementary Fig. 20(b) that when the moving

car is close to the receiving antenna, the wireless communication is reliable and the BER is stable at 10-5, whilst when the moving car is far away from the receiving antenna, the beam is no longer made towards the receiving antenna and the BER value is very high. In contrast, in the second experiment, it is observed in Supplementary Fig. 21(b) that the wireless communication is always reliable because the beam is always made towards the receiving antenna on the moving model car, and the BER value is stable at about 10^{-5}.

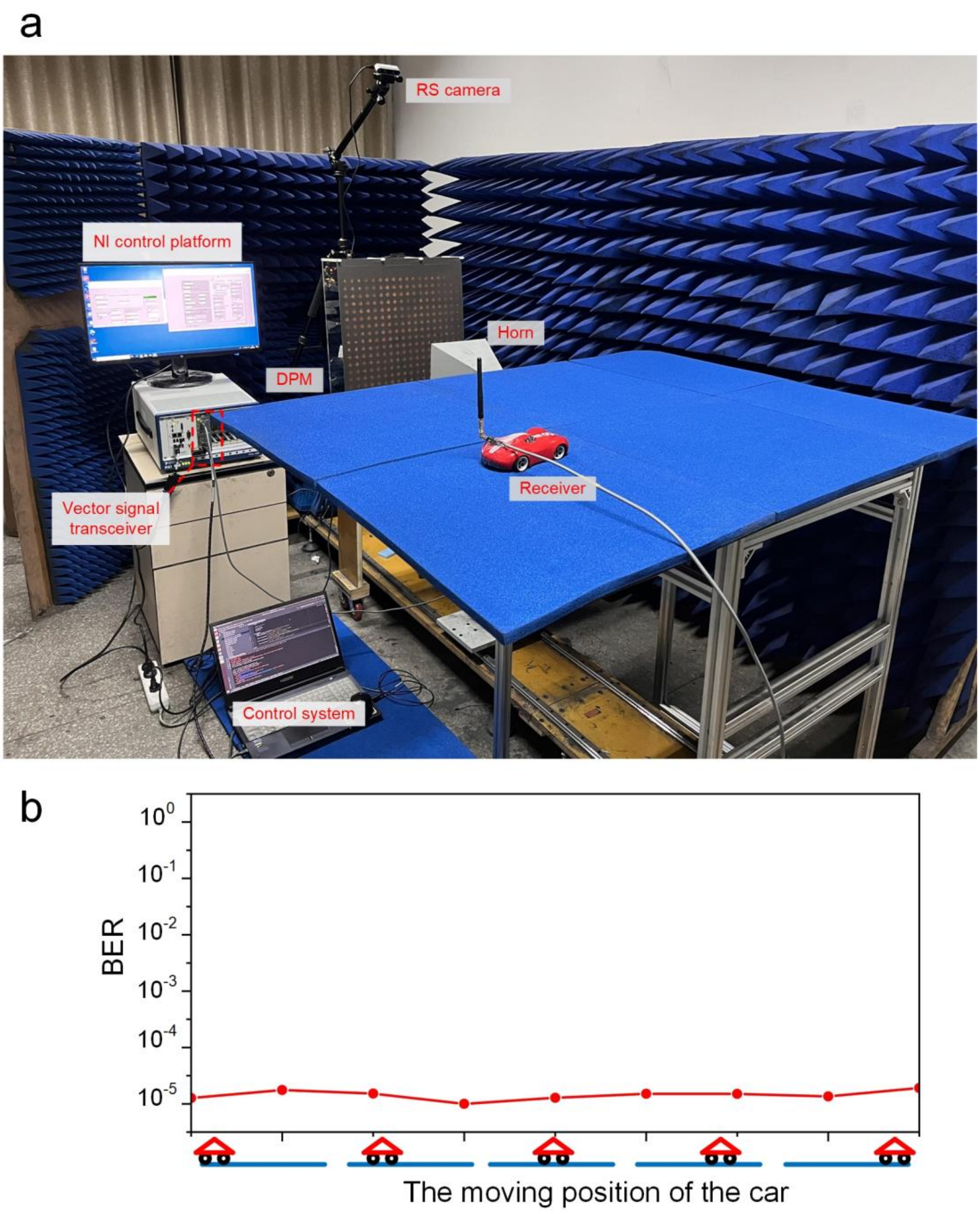

Supplementary Fig. 21 (a) Experimental setup of BER testing when the receiving antenna is fixed on the moving car. (b) The measured value of BER.

Supplementary Note 17. Discussion on how to solve the potential issue of interference from other actively communicating devices operating at a similar frequency

In a more practical environment, there are interference problems such as other communication devices in adjacent frequency bands. For the elimination of interference, the following will be explained from the programmable ability and dual-polarization of DPM itself, and some potential ways, such as by enhancing wireless sensing.

Firstly, we demonstrate that the DPM is able to solve the potential issue of interference from other actively communicating devices operating at a similar frequency. The reflected phase responses of the coding element and the calculated 2D far-field patterns are plotted in Supplementary Fig. 22 (a-c). When the phase difference between the two states ("00" and "10") of the element is 180° ± 37°, the far-field patterns at 5.7 to 5.9 GHz are quite good except for some acceptable increase of sidelobes and the center frequency is 5.8 GHz. In contrast, at a similar frequency outside the operating band, e.g., at 5.6 GHz and 6.0 GHz, the DPM can no longer create directive beam, as is shown in Supplementary Fig. 21(c). In view of this, we conclude that other actively communicating devices operating outside the band of 5.7-5.9 GHz cannot disturb the communication because they only result in very weak reflected EM energy towards the target.

Secondly, we demonstrate that the DPM can also depress in-band interference from devices other than the feeding source. The coding pattern of the DPM should be designed with regard to the position of the feeding source, so as to complete phase compensation and achieve efficient deflection towards the direction of target. For example, Supplementary Fig. 22 (d-f) give the coding patterns of 20° deflection when the feeding horn is at different positions. In Supplementary Fig. 22 (d) and (f), the horn is 300 mm away from the DPM and the incident angles (in terms of θ and φ) are (0°, 0°) and (45°, 0°) respectively. In Supplementary Fig. 22 (e), the horn is 600 mm away, and the incident angle is (0°, 0°). Blue patches denote OFF-state PIN diodes, and yellow patches denote ON-state PIN diodes. Clearly, the coding sequence of DPM is determined by the position of its feeding source and the required beam deflection. The same coding sequence creates different beam deflection for the external sources at different locations, even though the sources are in the operating frequency band of the DPM. Therefore, interference of external sources can be effectively depressed by the DPM itself.

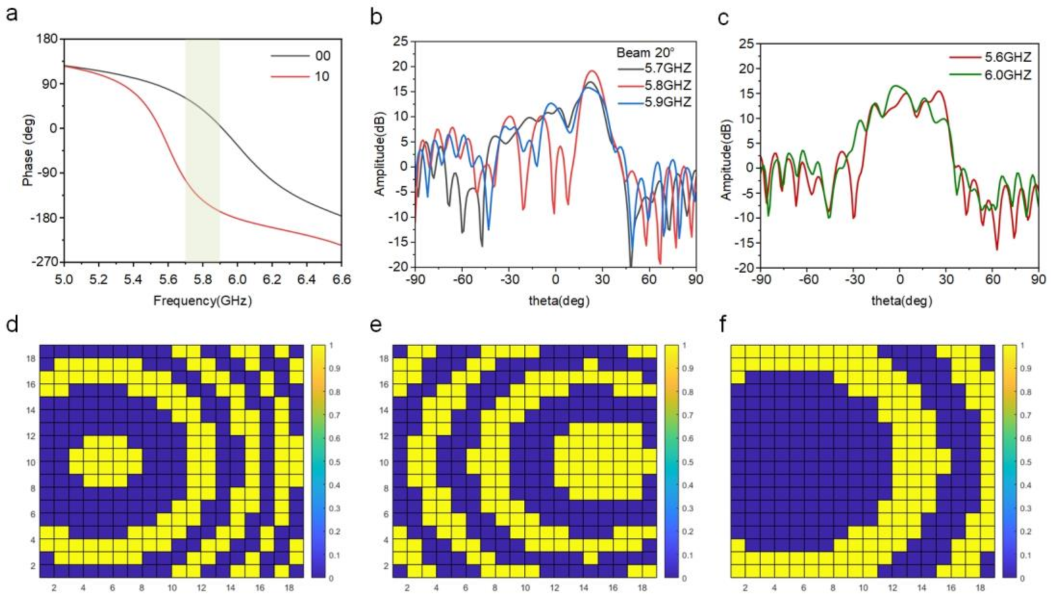

Supplementary Fig. 22 (a) The reflected phase responses of the coding element when the PIN diodes are switched ON and OFF in case of y-polarization. (b) Calculated 2D far-field results at 5.7 GHz, 5.8 GHz, and 5.9 GHz. (c) Calculated 2D far-field results at 5.6 GHz, and 6.0 GHz. (d-f) Digital coding schemes for beams towards 20° in the E-plane. Blue patches denote the OFF-state PIN diodes and yellow patches denote the ON-state ones. Different incident angles and distances correspond to different DPM coding patterns. In (d), the feeding horn is 300 mm away from the metasurface and the incident angle (theta, phi) is (0, 0). In (e) the horn is 600 mm away and the incident angle is (0, 0). In (f) it is 300 mm away and the incident angle is (45°, 0).

Next, we investigate the influence of interference when its power increases. In the commercial software CST, we established a rectangular waveguide as the interference source, named Port 2 as shown in Supplementary Fig. 23(a). Port 2 is about 600 mm away from the DPM with an incident angle of 15°. The feeding horn of DPM is 300 mm away and is named Port 1. The deflection angle (θ, φ) corresponding to the encoded pattern of DPM is (24°, 0°). We carried out six cases of simulation, in which the amplitudes of Port 1 and Port 2 are 1:0, 0:1, 1:1, 1:0.5, and 1:0.1 respectively, the last case is when the amplitudes of port 1 and port 2 are 1:0.1, the phase difference is 180 degrees, as given in Supplementary Fig. 23(b). From Supplementary Fig. 23(c, d) to observe the influence of interference sources on the beam in more detail. It can be seen from the results that when the energy of the interference source is low, for example, when the energy of the interference is one tenth of that of the feed, the influence on the main beam is neglectable. However, when the energy of the interference is much higher than that of the feed, the beam width and directivity are affected to some extent,

and the sidelobes are significantly higher. Therefore, the DPM can depress in-band interference with comparable power. And when the power of external interference is relatively high, we need to increase the feeding power of the DPM.

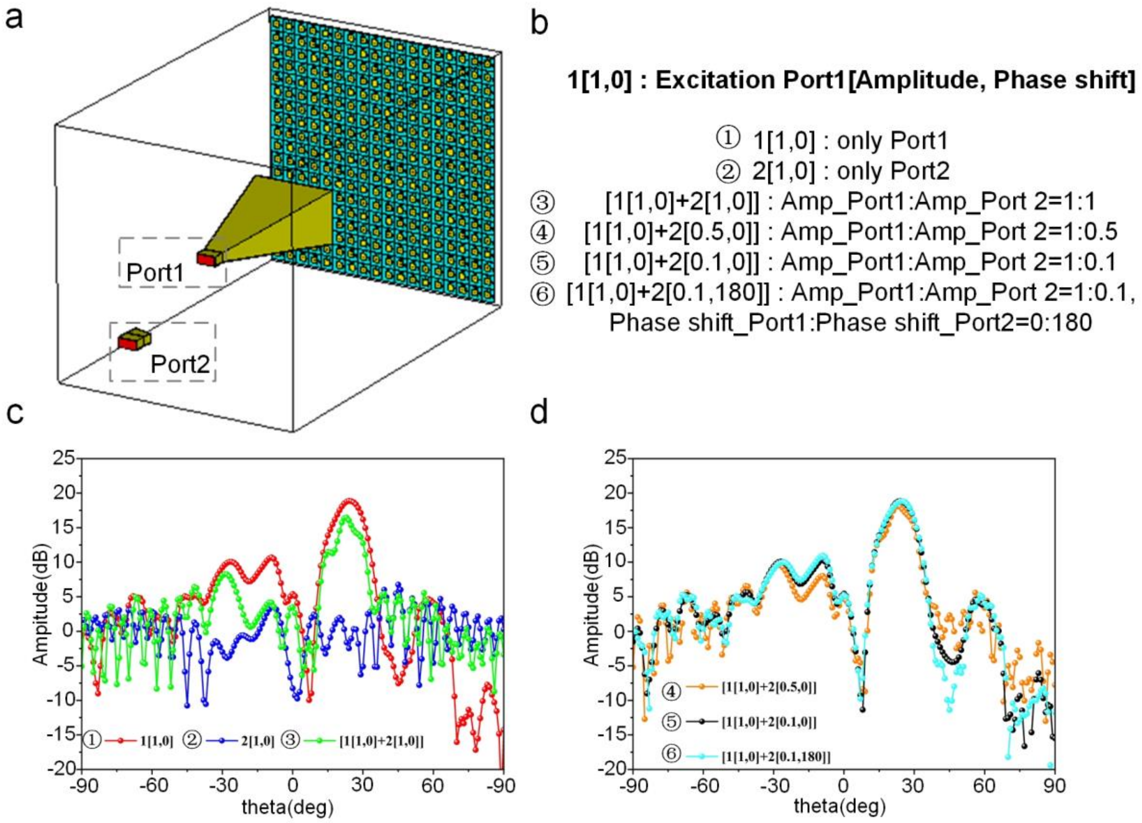

Supplementary Fig. 23 (a) Simulation model of the proposed DPM with two-port excitation. (b) A description of the different excitation amplitudes of the two ports. (c, d) the co-simulation far-field results under different excitations.

Moreover, dual-polarized DPM can help to depress the interference. In a dual-polarized DPM, each element can independently modulate the phases for different polarizations and reflect dual-polarized signals with high isolation. Each polarization is controlled in real time through an individual interface to FPGA. For the interference at a similar frequency, DPM is used to regulate the signals of the two polarization channels, and the better polarization channel is selected by observing the bit error rate received by the signals at the receiver. For the verification of dual-polarization performance (please refer to Supplementary Notes 4 and 15).

Below is some discussion of possible methods for same frequency interference elimination. Similar frequency interference is a problem in many scenarios such as weather radar, distorting radar variable estimation, etc. Several methods have been adopted and studied

by many scholars for solving the interference problem. For example: 1. setting an isolation board. 2. Design specific property of the transmitter or the receiver to eliminate interference. In addition, some filters are often adopted for solving interference problems. For example, adaptive notch filter[23], object-orientated spectral polarimetric (OBSPol) filter[24], and nonlinear filtering[25]. Sidelobe blanking[26] is also used in communication systems to mitigate interference.

In ref[27] and Supplementary Fig. 24, the wireless sensing system exploits the antenna pattern diversity and software programmability of the dynamic metasurface antennas (DMA) to achieve high-performance wireless sensing. A general framework for DMA-based wireless sensing, and demonstrate the feasibility and benefits of the DMA in sensing using custom hardware. A general deep learning framework for RF sensing in the IoT has been proposed[28]. A potential solution is to use the antenna pattern diversity of DMA to generate rich high-dimensional channel measurements, and simulate the influence of environmental dynamics on the received signal. In other words, for a given sensing application, people can learn a set of common features shared between different DMA antenna patterns. Due to the change of antenna pattern or environment, the learned features should be robust to signal diversity. Therefore, the receiver extracts the information of the interference signal through signal processing, and then feedback it to the reconfigurable metasurface to achieve an optimized coding pattern, so as to eliminate the interference of similar frequency.

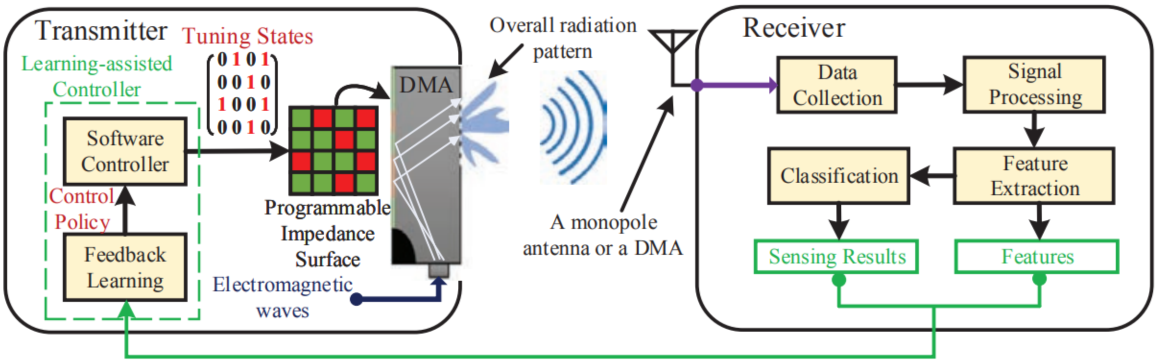

Supplementary Fig. 24 The end-to-end design of the DMA-based wireless sensing system[27]. © [2022] IEEE. Reprinted, with permission, from [G. Lan, M. F. Imani, P. d. Hougne, W. Hu, D. R. Smith and M. Gorlatova, "Wireless Sensing Using Dynamic Metasurface Antennas: Challenges and Opportunities," in IEEE Communications Magazine, vol. 58, no. 6, pp. 66-71, June 2020, doi: 10.1109/MCOM.001.1900696].

Supplementary Note 18. The energy consumption of the proposed design

We categorize the devices involved in this design and list their power consumption in Supplementary Table 4. The RS-camera and the control system (with a laptop included) are combined as a whole. We can monitor the power consumption on the laptop when the control system is in standby state (as shown in Supplementary Fig. 25 (b)) and when it is running the RS-camera for tracking tasks and sending FPGA instructions (as shown in Supplementary Fig. 25 (c)). The working state of DPM is mainly determined by the power supply of FPGA, so the power consumption of FPGA and DPM is displayed in one column. In the experiment of RF signal detection, the transmitter is a microwave signal generator (Keysight E8267D) and the detecting module is mainly composed of a detector AD8317 and a micro controller unit (MCU). In the experiment of real-time wireless transmissions, the image transmission module is responsible for most power consumption.

Considering that the theoretical calculation or self-displayed power may not be available in practice, we used a power detector DL333501 to test the energy consumption of the design, as given in Supplementary Table 4. DPM is powered by FPGA, so the power consumption sum of this part is 13.129 W, FPGA devices that consume less power can reduce this value even more. For the RS-camera and the control system, the displayed powers are 8 W and 27 W for the "standby" and "running" states, respectively, the camera is connected to the labtop by USB interface, no extra power supply, the values are recorded in Supplementary Table 4.

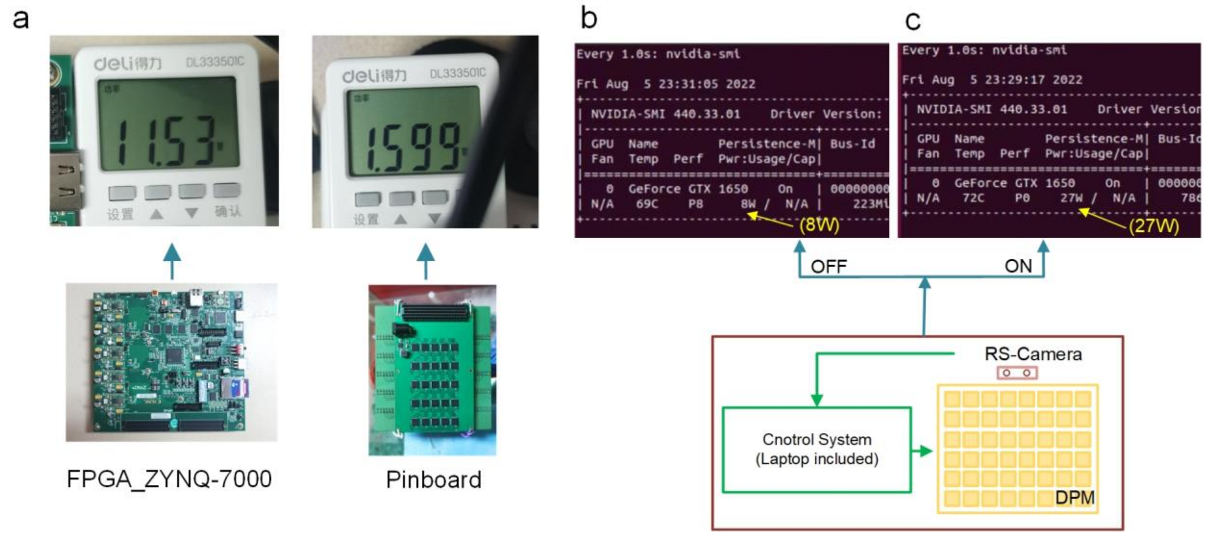

Supplementary Fig. 25 Energy consumption of the design. (a) The power detector DL333501 used to measure the power consumption of instruments in the design. The measured power consumptions of the main

instrument in use. (b, c) The power consumption displayed on the laptop when the control system is in "standby" state and "running" state. ON and OFF represent the "running" and "standby" states of the intelligent tracking algorithm, respectively.

Supplementary Table 4. Energy consumption of the design

Instruments	Implications	The value of the system
RS-camera and the control system	Standby state<br>Running state	**8 W**<br>**27 W**
FPGA with DPM	DPM is powered by FPGA, so the sum of FPGA and Pinboard is the power consumption of this part	**13.129 W**
Signal generator	-10 dBm	**Variable**
Detector	A detector AD8317 and MCU	**0.5×10^{-3} W**
Real-time wireless transmissions	The image transmission module	**5×10^{-3} W**

Next, we take into consideration of the change of performance when the target is located at different distances from the DPM under different transmitting powers. We aligned the target in the directions of θ=-30° and θ=20°and tested the received energy when it was 1.5 m and 3.0 m away from the DPM. Supplementary Fig. 26 (a, b) shows the schematic of the testing scenario, where the signal generator (SG) is connected to the horn as the transmitting source and the receiving antenna is connected to the spectrum analyzer (SA) to measure the power of the received signal. We measured the received power values when the target was 1.5 m and 3.0 m away from DPM and the transmitting power was set to -10 dBm, 0 dBm, 10 dBm and 20 dBm respectively, at the two directions of -30° and 20°. Supplementary Fig. 26 (c, d) plots the measured results. It is observed that with the same transmitting power, the received power decreases by an average of 2 dBm. As the transmitting power increases, the received power also increases by the same amount, as indicated by the curves in Supplementary Fig. 26 (c, d). In view of this, we conclude that the total energy consumption is also dependent on the power required by the receivers, the number of receivers, and the communication distance.

Some considerations could be involved in the future work to further reduce the power consumption. We can use a single FPGA development board connected to the camera to run the

target tracking algorithm and realize the task of sending voltage sequences to the DPM. In addition, the use of low power varactor and numerical control rheostat can also help to reduce the power consumption of the system.

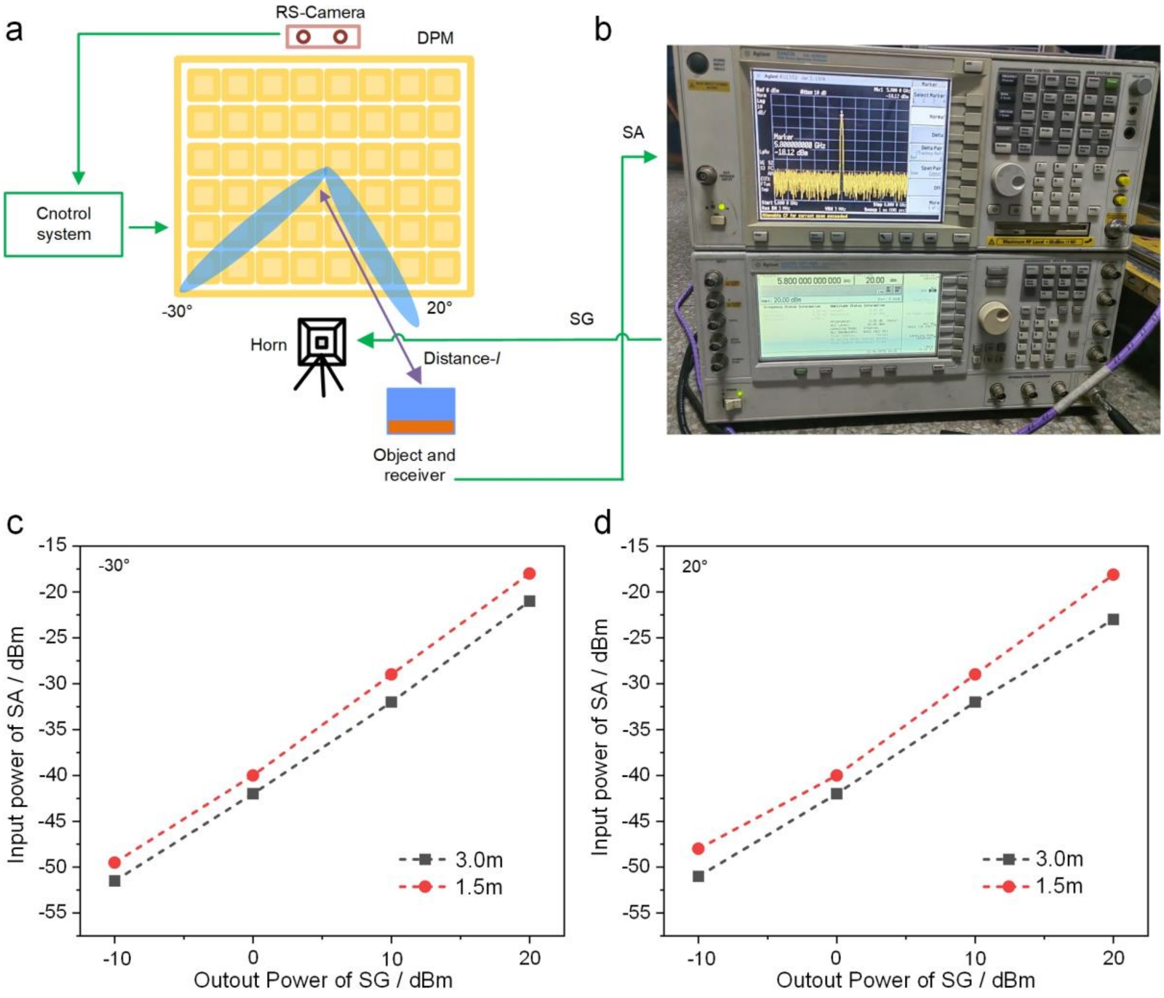

Supplementary Fig. 26 (a) Schematic of the experimental setup to measure the power of received signal. (b) The transmitting horn is connected to the signal generator (SG), and the receiving antenna is connected to the spectrum analyzer (SA). The power of received signal is plotted when the target is aligned in the directions of (c) -30° and (d) 20°.

Supplementary Note 19. The structure of the YOLOv4-tiny network used in this paper.

We divide the structure of YOLOv4-tiny network into three parts, as given in Supplementary Fig. 28-30. Supplementary Fig. 27 shows the connection order of the network.

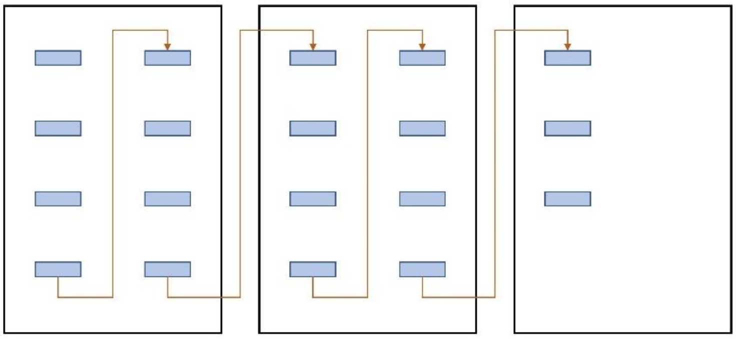

Supplementary Fig. 27 The connection order of the YOLOv4-tiny network. Part 1 and 2 are divided into left and right columns, the bottom of the left column is connected to the top of the right column, and Part 3 is the single column following by the bottom of the right column of Part 2.

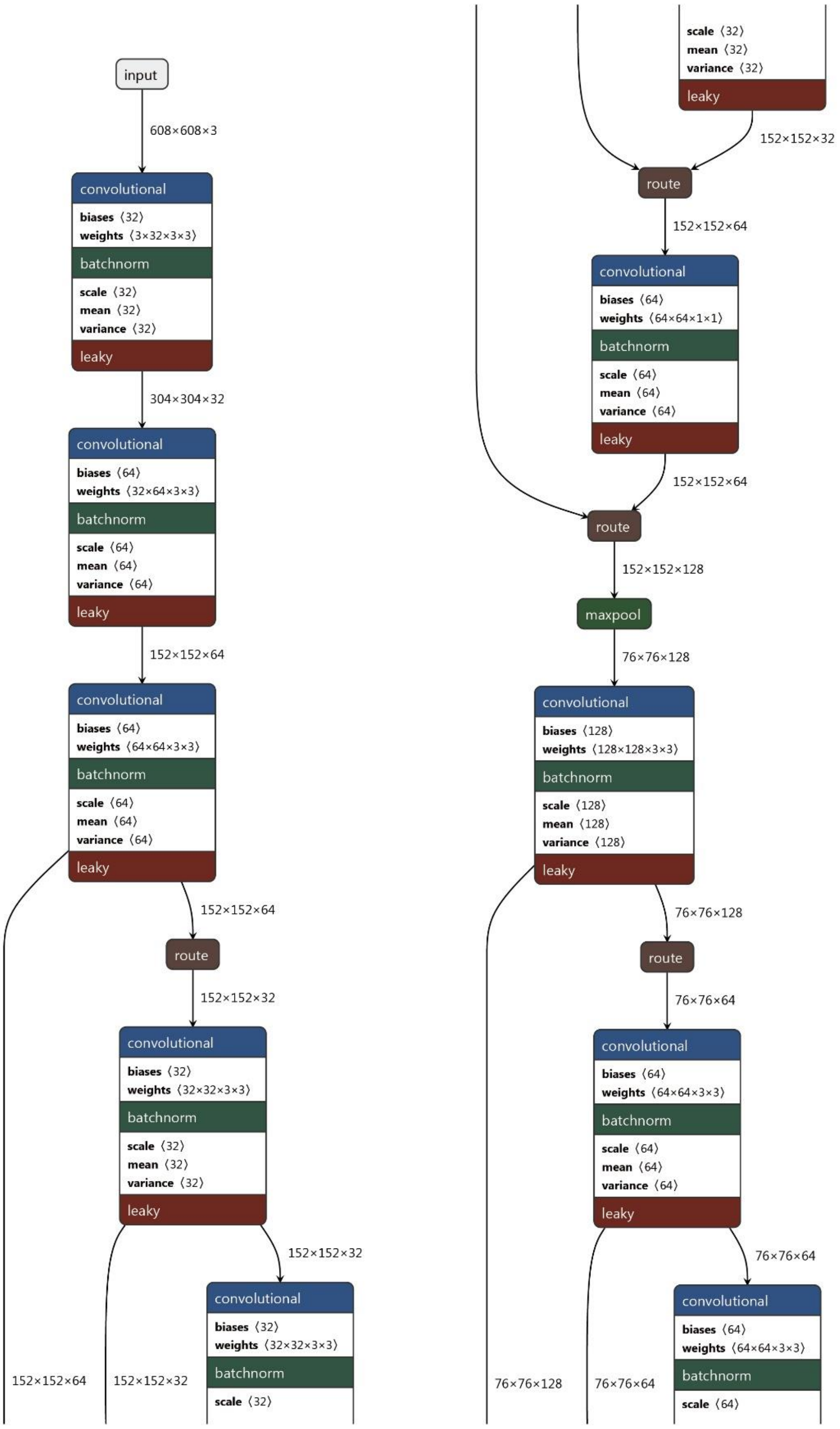

Supplementary Fig. 28 Part 1 of the structure of the YOLOv4-tiny network used in this paper.

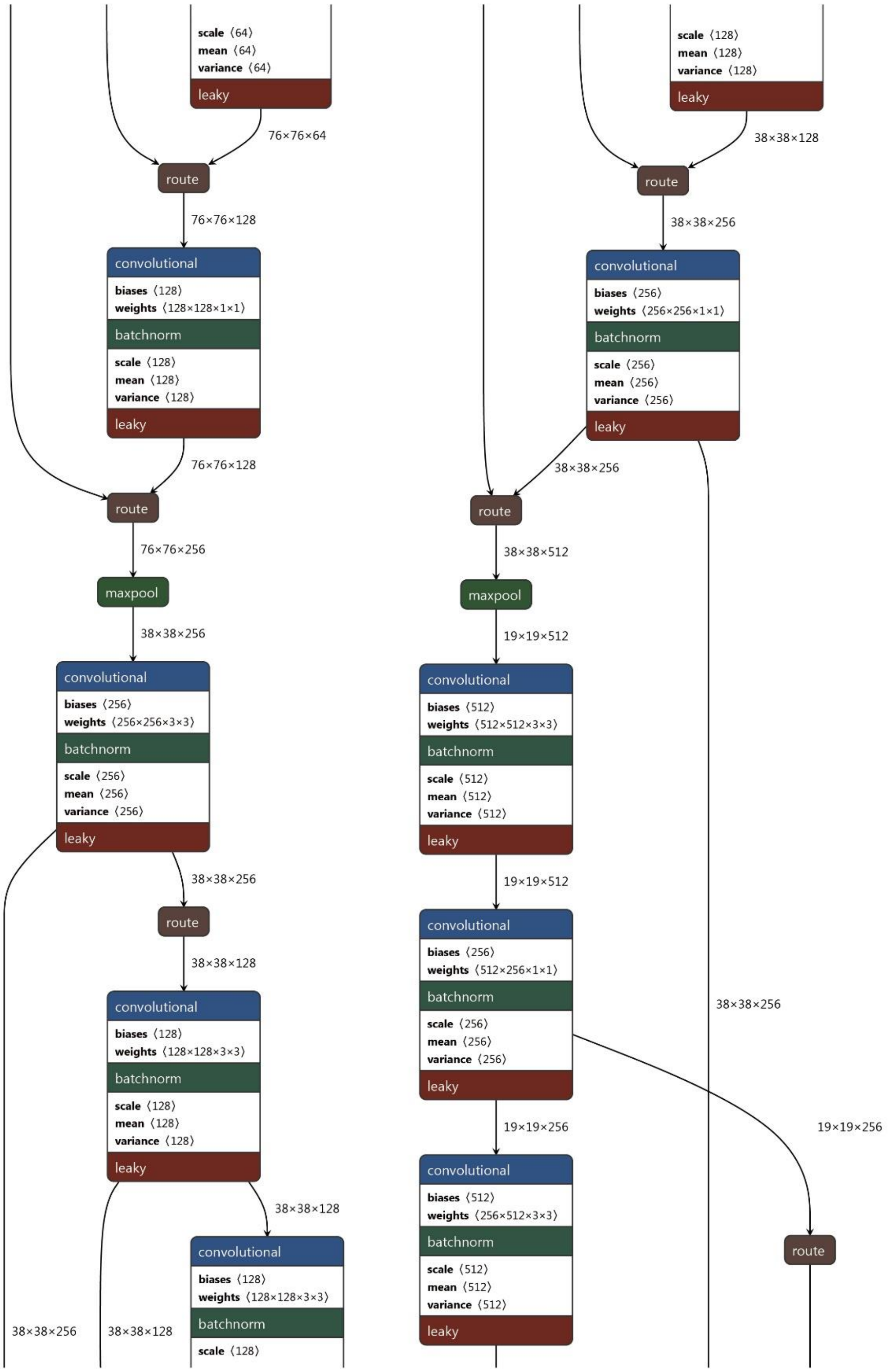

Supplementary Fig. 29 Part 2 of the structure of the YOLOv4-tiny network used in this paper.

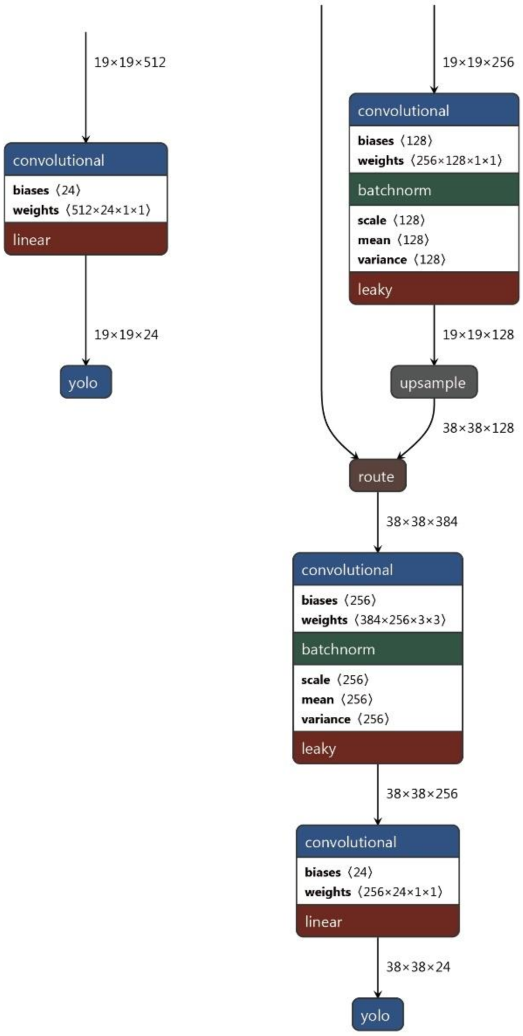

Supplementary Fig. 30 Part 3 of the structure of the YOLOv4-tiny network used in this paper.

Supplementary References

1. Molchanov, P., Tyree, S., Karras, T., Aila, T. & Kautz, J. Pruning Convolutional Neural Networks for Resource Efficient Transfer Learning. *CoRR* **abs/1611.06440**, (2016).
2. Redmon, J., Divvala, S., Girshick, R. & Farhadi, A. You Only Look Once: Unified, Real-Time Object Detection. in *2016 IEEE Conference on Computer Vision and Pattern Recognition (CVPR)* 779–788 (2016).
3. Redmon, J. & Farhadi, A. YOLO9000: Better, Faster, Stronger. in *2017 IEEE Conference on Computer Vision and Pattern Recognition (CVPR)* 6517–6525 (2017).
4. Redmon, J. & Farhadi, A. YOLOv3: An Incremental Improvement. *CoRR* **abs/1804.02767**, (2018).

5. Bochkovskiy, A., Wang, C.-Y. & Liao, H.-Y. M. YOLOv4: Optimal Speed and Accuracy of Object Detection. *CoRR* **abs/2004.10934**, (2020).
6. Wang, C.-Y., Bochkovskiy, A. & Liao, H.-Y. M. Scaled-YOLOv4: Scaling Cross Stage Partial Network. *CoRR* **abs/2011.08036**, (2020).
7. Geron, A. *Hands-on Machine Learning with Scikit-Learn and TensorFlow*. (2017).
8. Donelli, M., Caorsi, S., Natale, F., Pastorino, M. & Massa, A. Linear Antenna Synthesis with a Hybrid Genetic Algorithm. *Prog Electromagn Res* **49**, (2004).
9. Robinson, J. & Rahmat-Samii, Y. Particle swarm optimization in electromagnetics. *IEEE Transactions on Antennas and Propagation* **52**, 397–407 (2004).
10. B. Sheen, J. Yang, X. Feng, & M. M. U. Chowdhury. A Deep Learning Based Modeling of Reconfigurable Intelligent Surface Assisted Wireless Communications for Phase Shift Configuration. *IEEE Open Journal of the Communications Society* **2**, 262–272 (2021).
11. Jia, Y. *et al.* In Situ Customized Illusion Enabled by Global Metasurface Reconstruction. *Advanced Functional Materials* **32**, (2022).
12. Shan, T., Pan, X., Li, M. & Xu, S. Coding Programmable Metasurfaces Based on Deep Learning Techniques. *IEEE Journal on Emerging and Selected Topics in Circuits and Systems* **PP**, 1–1 (2020).
13. Li, S., Liu, Z., Fu, S., Wang, Y. & Xu, F. Intelligent Beamforming via Physics-Inspired Neural Networks on Programmable Metasurface. *IEEE Transactions on Antennas and Propagation* **70**, 1–1 (2022).
14. Qian, C. *et al.* Deep-learning-enabled self-adaptive microwave cloak without human intervention. *Nature Photonics* **14**, 383–390 (2020).
15. He, K., Zhang, X., Ren, S. & Sun, J. Deep Residual Learning for Image Recognition. *CoRR* **abs/1512.03385**, (2015).
16. Ioffe, S. & Szegedy, C. Batch Normalization: Accelerating Deep Network Training by Reducing Internal Covariate Shift. (2015).
17. Kingma, D. & Ba, J. Adam: A Method for Stochastic Optimization. *International Conference on Learning Representations* (2014).
18. Yang, H. *et al.* A programmable metasurface with dynamic polarization, scattering and focusing control. *Scientific Reports* **6**, 35692 (2016).

19. Nayeri, P. & Elsherbeni, A. *Reflectarray Antennas: Theory, Designs, and Applications*. *Reflectarray Antennas: Theory, Designs, and Applications* (2018).
20. Zhang, Y., Wang, C., Wang, X., Zeng, W. & Liu, W. FairMOT: On the Fairness of Detection and Re-identification in Multiple Object Tracking. *International Journal of Computer Vision* **129**, 3069–3087 (2021).
21. Wojke, N., Bewley, A. & Paulus, D. Simple online and realtime tracking with a deep association metric. in *2017 IEEE International Conference on Image Processing (ICIP)* 3645–3649 (2017).
22. Analog Devices. AD8317:1MHz to 10GHz, 55dB Log Detector/Controller Data Sheet. (2008). https://www.analog.com/cn/products/ad8317.html
23. Chen, G., Ren, Z., Li, Y. & Zhang, T. A Method of Same Frequency Interference Elimination Based on Adaptive Notch Filter. in *2009 International Workshop on Intelligent Systems and Applications* 1–4 (2009).
24. Yin, J., Hoogeboom, P., Unal, C. & Russchenberg, H. Radio Frequency Interference Characterization and Mitigation for Polarimetric Weather Radar: A Study Case. *IEEE Transactions on Geoscience and Remote Sensing* **60**, 1–16 (2022).
25. Arce, G. R. & Hasan, S. R. Interference term elimination of the discrete wigner distribution using nonlinear filtering. in *2000 10th European Signal Processing Conference* 1–3 (2000).
26. Shnidman, D. A. & Shnidman, N. R. Sidelobe Blanking with Expanded Models. *IEEE Transactions on Aerospace and Electronic Systems* **47**, 790–805 (2011).
27. Lan, G. *et al.* Wireless Sensing Using Dynamic Metasurface Antennas: Challenges and Opportunities. *IEEE Communications Magazine* **58**, 66–71 (2020).
28. Wang, X., Wang, X. & Mao, S. RF Sensing in the Internet of Things: A General Deep Learning Framework. *IEEE Communications Magazine* **56**, 62–67 (2018).
