# Supplementary material for: Intelligent metasurface system for automatic tracking of moving targets and wireless communications based on computer vision
Source: Nat Commun. 2023 Feb 22;14:989. doi: 10.1038/s41467-023-36645-3 (PMC9947232; doi:10.1038/s41467-023-36645-3)
Supplement: Supplementary file 3 — Description of Additional Supplementary Files [file 41467_2023_36645_MOESM3_ESM.pdf]

Description of Additional Supplementary Note

Supplementary Movie 1. The received energy measured by a vector network analyzer (VNA), when the receiving antenna is fixed in the moving path of the car.

Supplementary Movie 2. The performance of object detection algorithm in multiple similar targets and target occlusion scenarios.

Supplementary Movie 3. The received energy measured by a VNA, in multiple similar targets scenarios.

Supplementary Movie 4. The received energy measured by a VNA, when multiple targets of different categories exist.

Supplementary Movie 5. The performance of object detection algorithm under limited ambient light, or when the environment is completely dark.

Supplementary Movie 6. The performance of RF signal detection when (top) the RF signal detector is attached to the moving car and (bottom) the detector is placed in the middle of the moving path of the car.

Supplementary Movie 7. Real-time wireless transmissions when the target moves and the receiver is fixed.

Supplementary Movie 8. Real-time wireless transmissions when the receiver is attached to the moving target.

Permission file for Supplementary Figure 9. A permission file for Supplementary Figure 9.

Permission file for Supplementary Figure 24. A permission file for Supplementary Figure 24.
